# Targeting of HIF2-driven cachexia in kidney cancer

**DOI:** 10.1038/s41591-025-04054-2

**Published:** 2025-11-28

**Authors:** Muhannad Abu-Remaileh, Laura A. Stransky, Nikita Bhalerao, Nitin H. Shirole, Qinqin Jiang, Eddy Saad, Marc Machaalani, Sean M. Vigeant, Hilina Woldemichael, Charles Xu, Jing Lu, Hairong Wei, Zhihong Liu, William Sun, Kei Enomoto, Toni K. Choueiri, Jason R. Pitarresi, Steven A. Carr, Namrata D. Udeshi, William G. Kaelin

**Affiliations:** 1 Department of Medical Oncology, Dana-Farber Cancer Institute, Harvard Medical School, Boston, MA USA; 2 Broad Institute of MIT and Harvard, Cambridge, MA USA; 3 Division of Hematology–Oncology, Department of Medicine, University of Massachusetts Chan Medical School, Worcester, MA USA; 4 Department of Medicine, Beth Israel Deaconess Medical Center, Harvard Medical School, Boston, MA USA; 5 NiKang Therapeutics, Wilmington, DE USA; 6 Howard Hughes Medical Institute, Chevy Chase, MD USA

**Keywords:** Renal cancer, Targeted therapies, Cancer metabolism

## Abstract

Kidney cancer frequently causes paraneoplastic syndromes, including hypercalcemia and cachexia, but the underlying mechanisms are incompletely understood. The most common form of kidney cancer, clear cell renal cell carcinoma (ccRCC), is frequently caused by loss of the pVHL tumor suppressor protein and the resulting upregulation of the HIF2 transcription factor. We show that *PTHLH*, which resides on a ccRCC amplicon on chromosome 12p, is a direct HIF2 transcriptional target in ccRCC. Further, we show that the increased *PTHLH* expression is both necessary and sufficient for the induction of hypercalcemia and cachexia in preclinical orthotopic cell line tumor models. Consistent with these observations, two different allosteric HIF2 inhibitors, belzutifan and NKT2152, rapidly ameliorated hypercalcemia and cachexia in patients with ccRCC, including in some who did not exhibit objective tumor shrinkage. Our findings support prospective clinical studies to determine whether HIF2 inhibitors can be leveraged not only for tumor control, but also for the treatment of cancer-associated cachexia in renal cell carcinoma.

## Abstract (web summary)

In vivo experiments and clinical cohort analyses show that hypoxia-inducible factor 2 (HIF2)-induced parathyroid hormone-related protein (PTHrP) expression contributes to cachexia in the context of renal cell carcinoma (RCC). The pathway can be targeted by HIF2 inhibitors, including belzutifan, which may reduce cachexia in patients with RCC.

## Main

Kidney cancer is one of the ten most common cancers in the USA, with ~81,500 new cases and 14,000 deaths each year^1^. Worldwide, an estimated 175,000 people died of kidney cancer in 2020^2^. Although early stage kidney cancer can often be cured surgically, treating advanced stage kidney cancer and its complications remains a challenge^1^.

By far the most common form of kidney cancer is ccRCC^3^. The initiating molecular event in most ccRCCs is biallelic inactivation of the von Hippel–Lindau (*VHL*) tumor suppressor gene resulting from *VHL* mutations or *VHL* hypermethylation^4^. The *VHL* gene product, pVHL, is the substrate recognition subunit of an E3 ubiquitin ligase that targets the alpha subunits of the heterodimeric transcription factor hypoxia-inducible factor (HIF) for proteasomal degradation when oxygen is abundant. In *VHL* mutant ccRCCs, loss of pVHL function causes inappropriate accumulation of HIF and HIF target genes even under normoxic conditions^5^. In preclinical models, HIF2, the transcription factor formed by HIF2α and an aryl hydrocarbon receptor nuclear translocator (ARNT) protein, and not the canonical HIF family member HIF1α, drives ccRCC proliferation in vitro and in vivo^5,6^. Drugs that block the HIF2-responsive growth factor vascular endothelial growth factor (VEGF) or its receptor KDR (kinase-domain related) are now mainstays of ccRCC treatment^7^. Upregulation of HIF2-responsive endogenous retroviruses might also contribute to the responsiveness of ccRCCs to immunotherapy^8^. Allosteric HIF2 inhibitors were also recently shown to be active against a subset of heavily pretreated patients with ccRCC^9^. The first of these, belzutifan (Welireg), is now approved for the treatment of sporadic ccRCCs and tumors arising in patients with germline *VHL* mutations (VHL disease)^10,11^.

Kidney cancer is sometimes called ‘the internist’s tumor’ because some patients with kidney cancer first come to medical attention as they suffer from one of its many paraneoplastic complications. Examples of such complications include fever of unknown origin, elevated erythrocyte sedimentation rate, hypertension, hypercalcemia, anemia, polycythemia, abnormal liver function tests without metastases (remote hepatopathy or Stauffer’s syndrome), coagulopathy, altered glucose metabolism, galactorrhea, amyloidosis and Cushing’s syndrome^12–14^. Polycythemia in the setting of ccRCC is almost certainly caused by ectopic production of the HIF2-responsive growth factor erythropoietin^15^. The molecular basis for most of the remaining ccRCC paraneoplastic syndromes remains obscure.

Cachexia, a multifactorial systemic syndrome that involves crosstalk between multiple organ systems across the body and is characterized by involuntary loss of muscle mass and fat leading to >5% body weight reduction, is a common complication of chronic conditions such as heart failure, chronic obstructive pulmonary disease and cancer^16^. This syndrome substantially diminishes quality of life, impairs tolerance to anticancer therapies and accounts for up to 20% of cancer-related deaths^17^. Notably, up to 35% of patients with renal cell carcinoma (RCC) develop cachexia^13,14^.

Parathyroid hormone-related protein (PTHrP) has been implicated in cachexia caused by some cancers and by kidney failure^18–23^. Here we show that PTHrP, which is encoded by the HIF2-responsive gene *PTHLH*, is a critical driver of two paraneoplastic syndromes in ccRCC: cachexia and humoral hypercalcemia. Using preclinical and clinical models, we demonstrate that both syndromes can be ameliorated with clinical grade HIF2 inhibitors, underscoring the therapeutic potential of targeting HIF2-driven pathways in ccRCC-associated paraneoplastic syndromes.

## Results

### HIF2 inhibition reverses cachexia in ccRCC xenograft models

**Extended Data Fig. 1:**
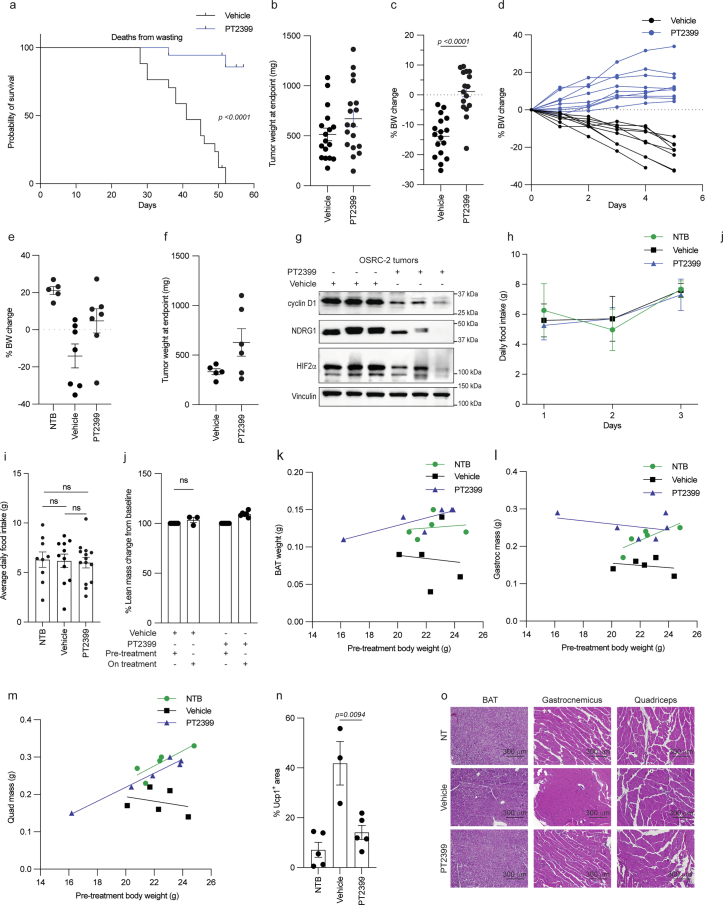
Supplemental data supporting the efficacy of PT2399 in treating cachexia induced by OSRC-2 xenografts. (**a**) Kaplan-Meier curve showing the probability of survival for OSRC-2 orthotopically implanted tumor-bearing mice treated with PT2399 (n = 18) or vehicle (n = 17). Deaths were attributed to wasting. Statistical analysis was performed using the log-rank test (p < *0.0001*). (**b**, **c**) (**b**) Tumor weight (**c**), body weight changes of mice in (*p* < *0.0001*) (**a**) (vehicle, n = 17; PT2399, n = 18). Each dot represents one mouse. (**d**) Individual trajectories of BW changes corresponding to Fig. 1b. (**e**–**j**) (**e**) Body weight change (NTB n = 5; vehicle n = 7; PT2399 n = 7), (**f**) tumor weight (vehicle n = 5; PT2399 n = 6) at endpoint. Each dot represents one mouse, (**g**) immunoblot analysis of representative OSRC-2 tumor lysates, (**h**) daily food intake in grams over 3 days (NTB n = 3; vehicle n = 4; PT2399 n = 5), (**i**) average daily food intake in grams plotted as individual daily measurements; each dot represents one day of intake from an individual mouse over 3 days (NTB 9 values from 3 mice, vehicle 11 values from 4 mice, PT2399 14 values from 5 mice), (**j**) percent fat mass change from baseline, measured by MRS body scan, in mice treated with PT2399 or vehicle (vehicle n = 3; PT2399 n = 6 at the on-treatment scan; group sizes reflect mice surviving to the scheduled scan under humane-endpoint criteria). (**k**–**o**) (**k**) Brown adipose tissue (BAT) mass versus pre-treatment body weight. (**l**) Gastrocnemius (Gastroc) muscle mass versus pre-treatment body weight. (**m**) Quadriceps (Quad) muscle mass versus pre-treatment body weight. Each dot represents an individual mouse; regression lines are shown for group comparisons. Two-sided ANCOVA adjusting for pre-treatment body weight revealed significantly increased BAT and Gastroc mass in PT2399-treated tumor-bearing mice compared to vehicle-treated controls (BAT: p = 0.023, 95% CI: 0.01–0.10 g; Gastroc: p = 0.001, 95% CI: 0.06–0.15 g). An interaction between treatment and pre-treatment body weight was detected for quadriceps mass, precluding ANCOVA analysis for that outcome, (**n**) quantification of Ucp1 positive area corresponding to Fig. 1j (NTB n = 5; vehicle n = 3; PT2399 n = 5, *p* = *0.0094*). Each dot represents one mouse, (**o**) Hematoxylin and eosin (H&E) staining of BAT, gastrocnemius muscle, and quadriceps muscle sections from tumor-bearing mice treated with vehicle or PT2399 compared to non-tumor bearing (NTB) mice. Data are presented as mean ± SEM. Statistical significance was assessed using unpaired two-tailed t-tests. Source data

HIF2 inhibitors hinder tumor growth and prolong survival in multiple preclinical ccRCC models, including both orthotopic cell line models and PDX models^24,25^. The HIF2 inhibitor PT2399 is a tool compound that is closely related to the approved drug belzutifan. We reported that PT2399 dramatically improved the survival of female nude mice bearing orthotopic tumors formed by the OSRC-2 *VHL*^−/−^ ccRCC cell line^26^ (Extended Data Fig. 1a). In this setting, however, PT2399 did not profoundly suppress tumor growth (Extended Data Fig. 1b). This was surprising because OSRC-2 cells are highly HIF2-dependent in vitro based on two- and three-dimensional cell culture proliferation assays^26^. Interestingly, we noticed that mice bearing OSRC-2 developed profound cachexia (Extended Data Fig. 1c), which ultimately required that they be euthanized, unless they were treated with PT2399.

**Fig. 1:**
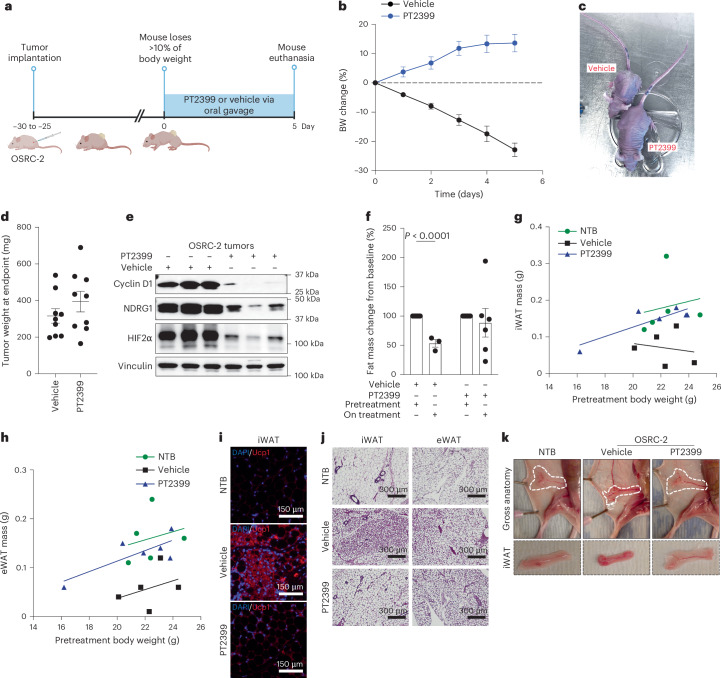
Successful treatment of cachexia caused by OSRC-2 xenografts with an HIF2 inhibitor. **a**, Treatment schema. **b**, Body weight (BW) changes over time in OSRC-2 tumor-bearing mice treated with PT2399 (*n* = 9) or vehicle (*n* = 9). **c**,**d**, Representative mouse images (**c**) and tumor weights (**d**) at study endpoint of mice from **b**. **e**, Immunoblot analysis of representative OSRC-2 tumor lysates at study endpoint in **d**. **f**, Percent fat mass change from baseline in OSRC-2 tumor-bearing mice treated with PT2399 or vehicle, measured by MRS body scan. Each dot represents one mouse (vehicle, *n* = 3; PT2399, *n* = 6 at the on-treatment scan; numbers reflect mice that survived to the scheduled scan under humane-endpoint criteria) (*P* < 0.0001). **g**,**h**, Scatterplots of iWAT (**g**) and eWAT (**h**) mass versus pretreatment body weight in NTB controls and OSRC-2 xenograft-bearing mice treated with vehicle or PT2399. Each dot represents an individual mouse. Group-specific linear regression lines are shown. Two-sided ANCOVA adjusting for pretreatment body weight revealed significantly higher iWAT and eWAT mass in PT2399-treated mice than vehicle-treated mice (iWAT, *P* = 0.020, 95% confidence interval (CI) 0.02–0.15 g; eWAT, *P* = 0.008, 95% CI 0.03–0.13 g). **i**,**j**, Immunofluorescent stain with DAPI and anti-Ucp1 for iWAT (**i**), and hematoxylin and eosin staining (**j**) of iWAT and eWAT sections from NTB, vehicle-treated and PT2399-treated groups. Representative images from three independent experiments with similar results. **k**, Gross anatomical images and isolated iWAT depots from NTB, vehicle-treated and PT2399-treated mice. For all panels, data are presented as mean ± s.e.m. unless otherwise indicated. Statistical significance was assessed using unpaired two-tailed *t*-tests; no multiple-comparison adjustments were applied. Illustrations in **a** created using BioRender.com. Source data

To study this observation prospectively, we performed additional OSRC-2 subcutaneous xenograft assays, monitoring body weight weekly. As expected, the tumor-bearing mice developed profound cachexia. Once the mice lost more than 10% of their body weight, they were randomized to PT2399 or vehicle by oral gavage (Fig. 1a). Mice treated with PT2399 rapidly regained weight, whereas the vehicle-treated mice continued to lose weight (Fig. 1b,c and Extended Data Fig. 1d). We weighed tumors removed at necropsies after 6 days of therapy or, in the case of one vehicle-treated mouse, after 5 days to prevent animal suffering. The PT2399-treated tumors were not smaller, and trended to be larger, than the vehicle-treated tumors, suggesting that the acute reversal of cachexia by PT2399 was pharmacodynamic and not due to a loss of tumor cells (Fig. 1d). Immunoblot assays confirmed other pharmacodynamic effects of PT2399, including downregulation of cyclin D1, NDRG1 and HIF2α protein levels itself^25–27^ (Fig. 1e). The latter has been described before and likely reflects destabilization of monomeric HIF2α when it is no longer bound to ARNT.

To study this phenomenon further, we undertook magnetic resonance spectroscopy (MRS) body scans of OSRC-2 tumor-bearing mice before and 17 days after treatment with PT2399 or vehicle. Once again, the vehicle-treated mice progressively lost weight, whereas PT2399-treated mice largely maintained their body weights despite comparable tumor burdens at necropsy (Extended Data Fig. 1e,f). Immunoblot assays further confirmed the pharmacodynamic effects of PT2399 (Extended Data Fig. 1g). Daily food intake did not differ between the two groups and was not diminished compared to nontumor-bearing mice (NTB) (Extended Data Fig. 1h,i). Body scans indicated that the weight loss in vehicle-treated mice was primarily due to reductions in fat mass, with lean mass largely being preserved (Fig. 1f and Extended Data Fig. 1j). To more accurately assess adipose and skeletal muscle tissue wasting while addressing known limitations of normalizing tissue weight to body weight, we plotted fat mass against pretreatment body weight and performed an analysis of covariance (ANCOVA). This analysis showed that PT2399 treatment preserved inguinal and epididymal white adipose tissue (iWAT and eWAT) compared with vehicle-treated controls (Fig. 1g,h). Similarly, brown adipose tissue and gastrocnemius muscle mass were also preserved in PT2399-treated mice (Extended Data Fig. 1k,l), while the assumption for ANCOVA was not met for quadriceps muscle (Extended Data Fig. 1m). The decreased WAT mass in vehicle-treated OSCR2 tumor-bearing mice was associated with upregulation of the thermogenic protein Ucp1 (Fig. 1i and Extended Data Fig. 1n), which causes energy dissipation as heat (thermogenesis) rather than through ATP generation^28^. Co-immunofluorescence of adipose tissue revealed that Ucp1, which marks white adipocytes that are converting to brown-like adipocytes with thermogenic properties (that is, ‘browning’), was reduced in the adipose of PT2399-treated mice (Fig. 1i and Extended Data Fig. 1n). In addition to Ucp1 induction, white adipocytes in vehicle-treated OSRC-2 tumor-bearing mice exhibited the hallmark switch from unilocular lipid-storing white adipocytes to multilocular thermogenic brown–beige adipocytes, a process that was blocked upon PT2399 treatment (Fig. 1j,k and Extended Data Fig. 1o). Collectively, these results suggest that cachexia in this model is largely caused by increased calorie utilization rather than decreased calorie intake and that PT2399 treatment blocks the induction of thermogenic gene programs in adipocytes.

### Proteomic profiling identifies PTHrP as a HIF2-responsive secreted factor

**Fig. 2:**
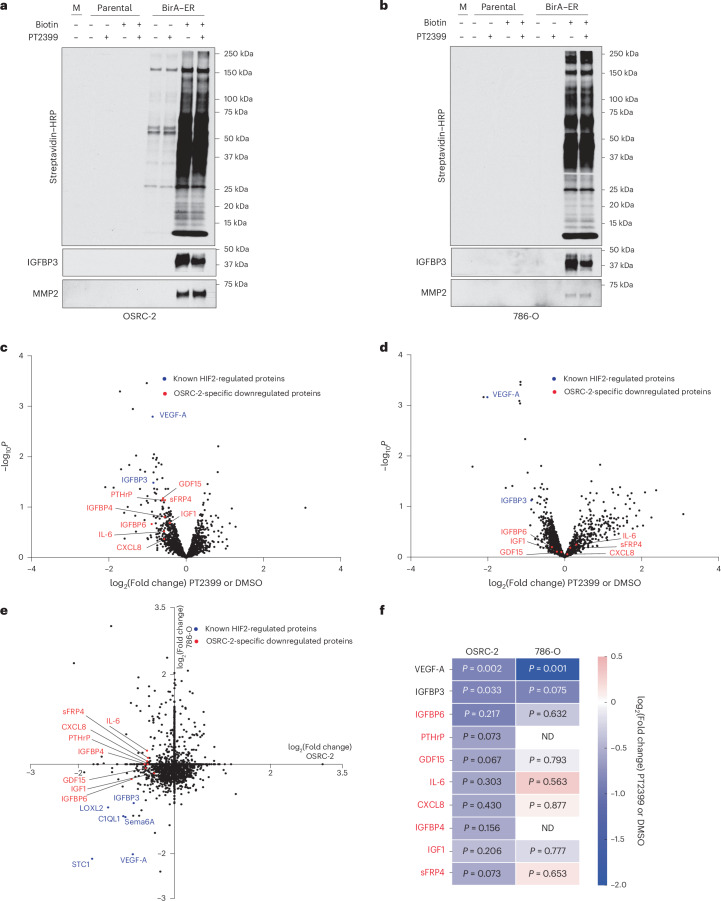
Identification of HIF2-responsive proteins secreted by OSRC-2 cells. **a**,**b**, Parental and BirA–ER OSRC-2 cells (**a**) or parental and BirA–ER 786-O cells (**b**) treated, where indicated, with biotin and/or 2 μM PT2399 for 48 h. Secreted proteins from conditioned media (or unconditioned media, ‘M’) were captured with SA, resolved by sodium dodecyl sulfate–polyacrylamide gel electrophoresis, transferred to nitrocellulose and detected with streptavidin–horseradish peroxidase or by immunoblotting with anti-IGFBP3 or anti-MMP2 antibodies. **c**,**d**, Volcano plots of −log_10_*P* versus log_2_(fold change) for proteins secreted by OSRC-2 cells (**c**) or 786-O cells (**d**) treated with PT2399 compared with DMSO (*n* = 2 biologically independent experiments). *P* values were calculated using two-sided moderated *t*-tests. **e**, Scatter plot comparing log_2_(fold change) values for secreted proteins regulated by PT2399 in OSRC-2 versus 786-O cells, showing overlapping known HIF2-regulated proteins in blue and OSRC-2-specific downregulated proteins in red. **f**, Heatmap displaying the log_2_(fold change) for proteins secreted by OSRC-2 and 786-O cells treated with PT2399 versus DMSO control. *P* values indicate statistical significance for each protein and were calculated using two-sided moderated *t*-tests (*n* = 2 biologically independent experiments). Exact *P* values are shown for each protein. Proteins in red appeared to be more sensitive to PT2399 in OSRC-2 cells than in 786-O cells. HRP, horseradish peroxidase; ND, nondectected proteins. Source data

To ask how, mechanistically, OSRC-2 cells induce cachexia, we introduced a promiscuous biotin ligase (BirA) fused to an endoplasmic reticulum targeting signal (ER) into OSRC-2 cells and, as a control, 786-O *VHL*^−/−^ ccRCC cells; 786-O cells do not induce profound cachexia in mouse xenograft assays. The Bir–ER fusion has been used by others to biotinylate secreted proteins that can then be captured using streptavidin agarose (SA) and identified by mass spectrometry (MS)^29^. In pilot experiments, we confirmed that cells expressing BirA–ER and treated with biotin secreted biotinylated proteins. To do so, proteins recovered from conditioned media using SA were eluted by boiling in sodium dodecyl sulfate-containing sample buffer, resolved by sodium dodecyl sulfate–polyacrylamide gel electrophoresis, and transferred to nitrocellulose filters that were probed with streptavidin–horseradish peroxidase (Fig. 2a,b). Streptavidin blots of conditioned media showed that cells expressing BirA–ER biotinylated a broad range of secreted proteins compared with conditioned media from cells lacking BirA–ER. The background signal in the BirA–ER OSRC-2 cells in the absence of supplemental biotin likely reflects that these cells, in contrast to the BirA–ER 786-O cells, were grown in RPMI media that contains low levels of biotin. Immunoblotting confirmed that known secreted proteins IGFBP3 and MMP2 were biotinylated in a BirA–ER-dependent manner in both cell lines.

To identify biotinylated proteins secreted by OSRC-2 and 786-O cells, we treated these cells (parental), as well as BirA–ER versions of these cells, with PT2399 or DMSO for 48 h, captured secreted proteins with SA, and identified secreted proteins by liquid chromatography tandem mass spectrometry (LC–MS/MS) using tandem mass tagging (TMT)-based quantification. We identified multiple PT2399-responsive secreted growth factors, including many that were shared across the two cell lines and some, such as VEGF-A, that are usually present in low (pM) abundance (Fig. 2c,d).

**Extended Data Fig. 2:**
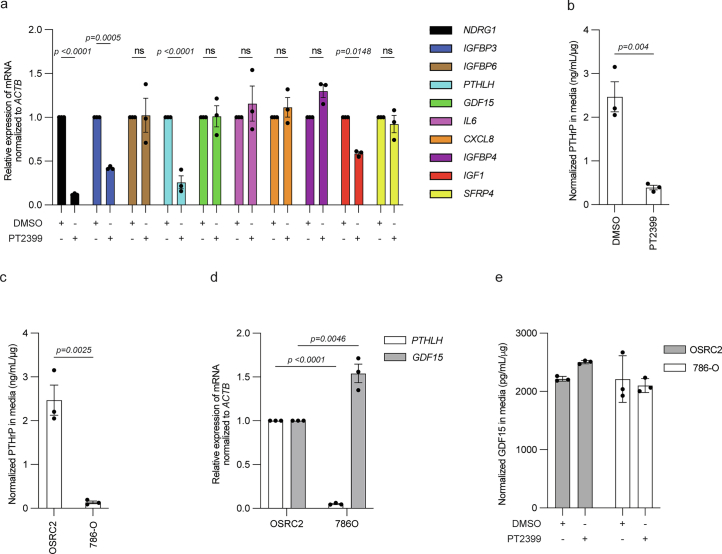
Validation of HIF2-responsive secreted proteins in OSRC-2 and 786-O cells. (**a**) mRNA levels for selected HIF2-responsive genes (*NDRG1* (*p* < *0.0001*)*, IGFBP3* (*p* = *0.0005*)*, IGFBP6, PTHLH* (*p* < *0.0001*)*, GDF15, IL6, CXCL8, IGFBP4, IGF1* (*p* = *0.0148*)*, SFRP4*) in OSRC-2 cells treated with 2 μM PT2399 or DMSO for 48 h. Each mRNA was normalized to *ACTB* and then to the DMSO value for that mRNA (n = 3). (**b**, **c**). PTHrP levels in media conditioned by OSRC-2 cells treated with 2 μM PT2399 or DMSO for 48 h (*p* = *0.004*) (**b**) or by OSRC-2 cells compared to 786-O cells (*p* = *0.0025*) (**c**) (n = 3). PTHrP levels were normalized to total cellular protein (ng/mL/μg). (**d**) Relative mRNA expression of *PTHLH* (*p* < *0.0001*) and *GDF15* (*p* = *0.0046*) in OSRC-2 and 786-O cells normalized to *ACTB* and then to the OSRC-2 value for that mRNA (n = 3). (**e**) GDF15 levels in media conditioned by OSRC-2 or 786-O cells treated with 2 μM PT2399 or DMSO for 48 h (n = 3). GDF15 levels were normalized to the total cellular protein (pg/mL/μg). Data are presented as mean ± SEM. Statistical significance was assessed using unpaired two-tailed t-tests or two-way ANOVA.

We focused on a short list of secreted proteins that were repressed by PT2399 in OSRC-2 cells but were not repressed, or far less repressed, by PT2399 in 786-O cells (proteins in red in Fig. 2e,f). Among these were PTHrP, GDF15 and interleukin-6, all of which have been linked to cachexia in various settings^18,19,30,31^. We confirmed that *PTHLH* and its protein product, PTHrP, are repressed by PT2399 in OSRC-2 cells and highly expressed in OSRC-2 cells relative to 786-O cells (Extended Data Fig. 2a–d). By contrast, *GDF15* messenger RNA and secreted protein levels were not repressed by PT2399 in OSRC-2 cells (Extended Data Fig. 2a,e). Moreover, GDF15 levels in OSRC-2 cell-conditioned media were comparable to the levels observed for 786-O cell-conditioned media, and normalized *GDF15* mRNA levels were higher in 786-O cells than in OSRC-2 cells (Extended Data Fig. 2d,e). *IL6* mRNA levels were similarly not repressed by PT2399 (Extended Data Fig. 2a). We therefore focused on PTHrP.

### *PTHLH* is a direct HIF2 target gene in kidney cancer cells

**Fig. 3:**
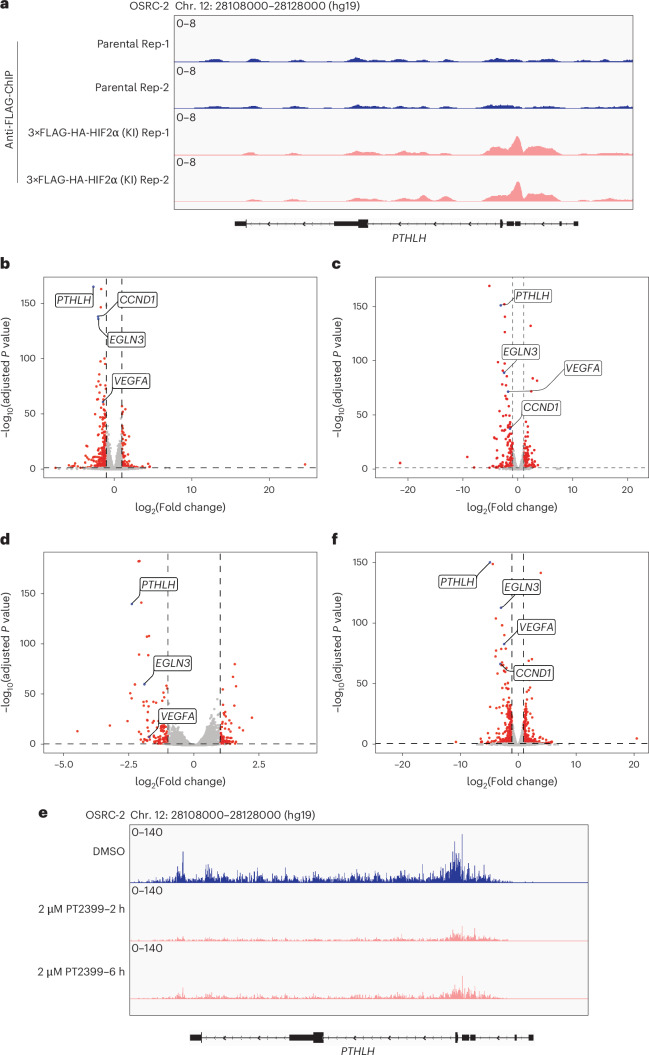
*PTHLH* is a direct HIF2 target gene in OSRC-2 cells. **a**, Anti-FLAG ChIP–seq tracks (two biological replicates) at the *PTHLH* locus in OSRC-2 cells with parental and 3×FLAG-HA-HIF2α knock-in (KI) cells. The *y* axis represents ChIP–seq read intensity, and the *x* axis corresponds to the chromosomal region chr. 12: 28108000–28128000 based on the hg19 human genome assembly. **b**,**c**, Volcano plots of RNA-seq data comparing OSRC-2 cells treated with 2 μM PT2399 or DMSO for 24 h (**b**) or infected to express sg*EPAS1* or sgCtrl (**c**). *PTHLH* and selected HIF2 target genes are highlighted in blue. Differential expression was computed using DESeq2 (two-sided Wald test; *n* = 3 biologically independent experiments), and *P* values were adjusted for multiple testing with the Benjamini–Hochberg FDR. **d**, Volcano plot of PRO-seq data from OSRC-2 cells treated with 2 μM PT2399 or DMSO for 2 h. Analysis performed with DESeq2 (two-sided Wald test; *n* = 3), with Benjamini–Hochberg FDR adjustment. **e**, PRO-seq tracks at the *PTHLH* locus in OSRC-2 cells treated with DMSO or 2 μM PT2399 for 2 and 6 h. The *y* axis represents transcriptional read intensity (0–140 arbitrary units) and the *x* axis corresponds to the chromosomal region chr. 12: 28108000–28128000 based on the hg19 human genome assembly. **f**, Volcano plot of polysome-seq data from OSRC-2 cells treated with 2 μM PT2399 or DMSO for 72 h. DESeq2 analysis as above (two-sided Wald test; *n* = 3, with Benjamini–Hochberg FDR adjustment). Data are presented as log_2_(fold change) versus −log_10_(adjusted *P* value). For volcano plots, statistical thresholds for significance were set at *P* ≤ 0.05 and *n* = 3. Dashed lines in the volcano plots indicate the thresholds for statistical significance (*P* = 0.05, log_2_(fold change) = ±1). The adjusted *P* value for *PTHLH* was zero but was set slightly above the smallest nonzero adjusted *P* value of the other genes for graphical purposes.

**Extended Data Fig. 3:**
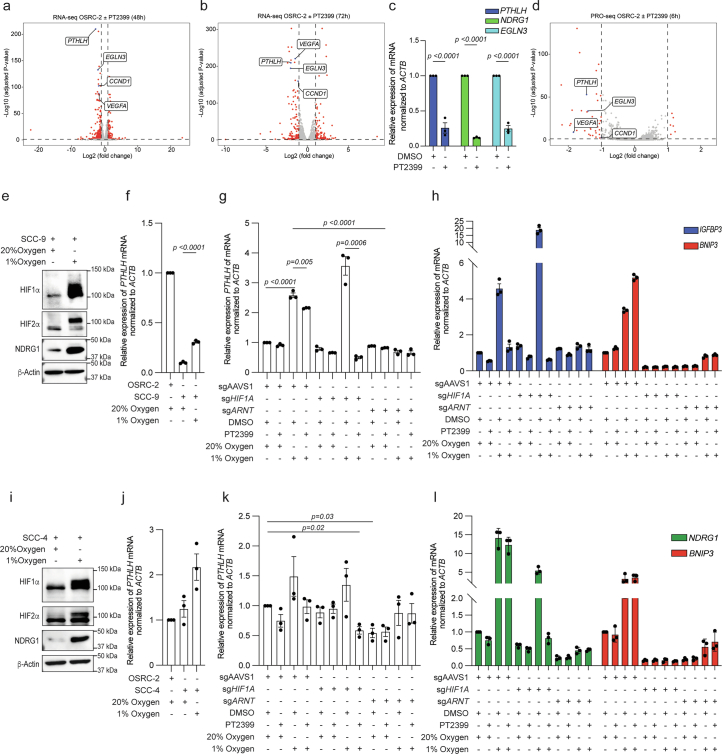
Extended analysis of *PTHLH* as a HIF2 target in OSRC-2 cells. (**a** and **b**) Volcano plots of RNA-seq data comparing OSRC-2 cells treated with 2 μM PT2399 or DMSO for 48 h (**a**) or for 72 h (**b**). PTHLH and key HIF2 target genes, including *VEGFA, EGLN3*, and *CCND1*, are highlighted in blue. Differential expression was computed using DESeq2 (two-sided Wald test; n = 3 biologically independent experiments), with Benjamini–Hochberg FDR adjustment. (**c**) Relative mRNA expression of *PTHLH, NDRG1*, and *EGLN3* in OSRC-2 cells treated with PT2399 or DMSO. Each mRNA was normalized to *ACTB* and then to the DMSO value for that mRNA (n = 3, *p* < *0.0001* for all genes). (**d**) Volcano plot of PRO-seq data from OSRC-2 cells treated with PT2399 or DMSO for 6 h, highlighting early transcriptional downregulation of *PTHLH, VEGFA, EGLN3*, and *CCND1*. DESeq2 analysis (two-sided Wald test; n = 3, with Benjamini–Hochberg FDR adjustment). (**e**, **f** and **i**, **j**) Immunoblot analysis (**e** and **i**) and qRT-PCR (**f** and **j**) of SCC-9 cells (**e** and **f**) or SCC-4 (**i** and **j**) incubated under 20% or 1% oxygen for 48 h. *PTHLH* mRNA was normalized to *ACTB* and then to the OSRC-2 value (n = 3). (**g**, **h** and **k**, **l**) Relative mRNA expression of *PTHLH (****g*** *and* ***k****)*, or the HIF2 target gene *IGFBP3* and the HIF1α target gene *BNIP3 (****h*** *and* ***l****)*, in SCC-9 cells (**g** and **h**) or SCC-4 (**k** and **l**) edited with sgAAVS1, sg*HIF1A*, or sg*ARNT*, treated with PT2399 or DMSO, and incubated under 20% or 1% oxygen levels for 48 h. Each mRNA was normalized to *ACTB* and then to the sgAAVS1-DMSO-20% oxygen value for that mRNA (n = 3, exact p values are indicated on the figure panels). Data are presented as mean ± SEM. Statistical significance was assessed using unpaired two-tailed t-tests and two-way ANOVA. For volcano plots, thresholds for significance were set at P ≤ 0.05, and n = 3. The adjusted p-value for *PTHLH* was zero but was set slightly above the smallest non-zero adjusted p-value of the other genes for graphical purposes. Source data

*PTHLH* has been reported to be HIF-responsive in various settings, including ccRCC, and to have a *cis*-acting HIF-binding site^24,25,32,33^. We confirmed the presence of the latter in chromatin immunoprecipitation sequencing (ChIP–seq) assays undertaken with OSRC-2 cells, wherein a FLAG-HA epitope tag was or was not appended to the endogenous HIF2α open reading frame (ORF) using CRISPR homology-directed repair (HDR)^27^ (Fig. 3a). We confirmed that *PTHLH* was among the most HIF2-responsive genes in OSRC-2 cells in steady-state RNA sequencing (RNA-seq) experiments done with OSRC-2 cells treated with PT2399 (Fig. 3b and Extended Data Fig. 3a,b) or after CRISPR KO of *EPAS*1 (Fig. 3c), which encodes HIF2α. We validated these RNA-seq findings by quantitative PCR with reverse transcription (RT–qPCR) (Extended Data Fig. 3c). In precision run-on sequencing (PRO-seq) experiments *PTHLH* transcription was dramatically reduced, as determined by RNA polymerase II recruitment, within 2 h of PT2399 treatment (Fig. 3d,e and Extended Data Fig. 3d). Polysome sequencing (polysome-seq) analysis of OSRC-2 cells treated with PT2399 for 72 h revealed significant translational downregulation of *PTHLH* (Fig. 3f). Therefore, *PTHLH* appears to be a direct HIF2 target in OSRC-2 cells.

Hypercalcemia driven by PTHrP has been described in other cancers, including squamous cell cancers of the head, neck and lungs^34^. We observed that *PTHLH* expression in the head and neck cancer cell line SCC-9 was induced by hypoxia (Extended Data Fig. 3e,f). In this setting, HIF1α and HIF2α cooperatively regulated *PTHLH* because *PTHLH* expression in SCC-9 was blocked by CRISPR-based inactivation of ARNT or by treatment with PT2399 if they were engineered to also lack HIF1α (Extended Data Fig. 3g,h). Qualitatively similar results were seen with a second head and neck cancer line, SCC-4 (Extended Data Fig. 3i–l). Therefore, HIF likely regulates *PTHLH* in cancers beyond kidney cancer.

### PTHrP is necessary for paraneoplastic cachexia and hypercalcemia in ccRCC

**Fig. 4:**
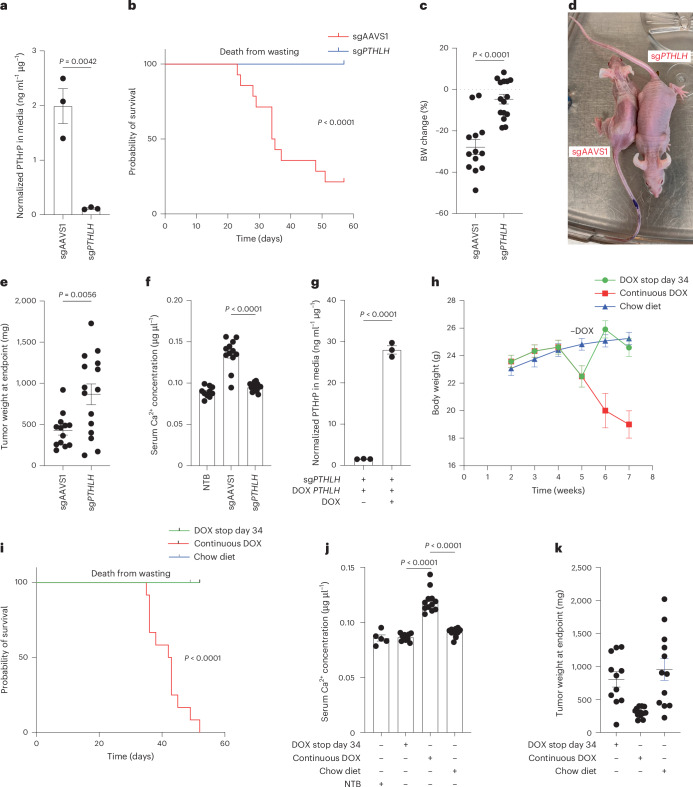
*PTHLH* is necessary for the induction of cachexia by OSRC-2 tumors. **a**, PTHrP levels in conditioned media from OSRC-2 cells infected to express sg*PTHLH* or sgAAVS1, normalized to total cellular protein (*n* = 3, *P* = 0.0042). **b**, Kaplan–Meier survival curve for mice with OSRC-2 tumors infected to express sg*PTHLH* (*n* = 15) or sgAAVS1 (*n* = 14) (*P* < 0.0001). **c**–**f**, Body weight changes (*P* < 0.0001) (**c**), representative mouse images (**d**), tumor weights (*P* = 0.0056) (**e**) and serum calcium levels (*P* < 0.0001) (**f**) at study endpoint of mice from **b**. Each dot represents an individual mouse. For body weight change and tumor weight: sgAAVS1, *n* = 13; sg*PTHLH*, *n* = 15. For serum calcium: sgAAVS1, *n* = 12; sg*PTHLH*, *n* = 15; NTB, *n* = 10. **g**, PTHrP levels in conditioned media from OSRC-2 sg*PTHLH* cells infected to express a DOX-inducible, sgRNA-resistant, *PTHLH* cDNA and grown in the presence or absence of DOX, normalized to total cellular protein (*n* = 3, *P* < 0.0001). **h**,**i**, Body weights (**h**) and Kaplan–Meier survival curves (*P* < 0.0001) (**i**) of mice bearing tumors formed by OSRC-2 cells from **g**. The mice were continuously fed DOX (*n* = 12) or never fed DOX (chow diet; *n* = 12) or had DOX stopped 34 days after cell implantation (*n* = 11). **j**,**k**, Serum calcium (*P* < 0.0001) (**j**) and tumor weights (**k**) at the endpoint in **i**. Continuous DOX (*n* = 12), chow diet (*n* = 12), DOX withdrawal (*n* = 11) and NTB (*n* = 5 for serum calcium). Each dot represents one mouse. Statistical analysis for Kaplan–Meier survival curve was performed using the log-rank test. For other panels, data are presented as mean ± s.e.m. Statistical significance was assessed using unpaired two-tailed *t*-tests.

**Extended Data Fig. 4:**
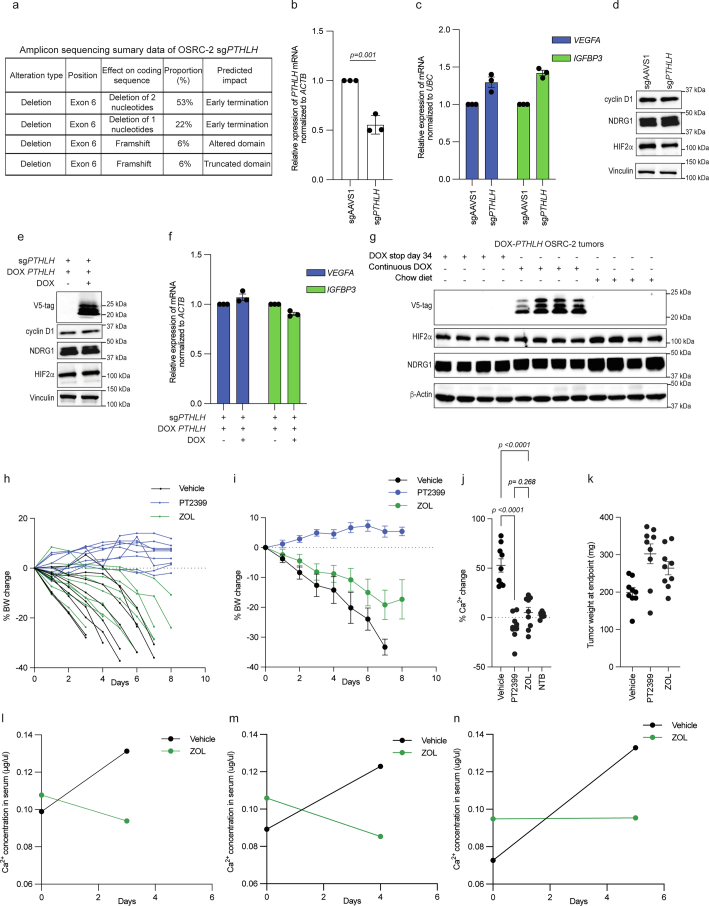
Characterization of OSRC-2 Cells after CRISPR-based editing with *PTHLH* sgRNA and Rescue with sgRNA-resistant *PTHLH* cDNA and Effects of Zoledronic Acid Treatment on Hypercalcemia and Cachexia. **a**) Amplicon sequencing data summary for OSRC-2 cells subjected to CRISPR editing with a *PTHLH* sgRNA. (**b**–**d**). qRT-PCR (**b** and **c**) and immunoblot analysis (**d**) of OSRC-2 cells that underwent CRISPR with a *PTHLH* sgRNA or AAVS1 sgRNA. In (**b** and **c**) mRNA levels were normalized to *ACTB* and then to the *ACTB*-normalized (**b**) or *UBC*-normalized AAVS1 sgRNA cell value for that mRNA (n = 3, *p* = *0.001* for (**b**)). (**e**–**g**) immunoblot (**e** and **g**) and qRT-PCR (**f**) analysis of OSRC-2 sgPTHLH cells infected to express a DOX-inducible, sgRNA-resistant, *PTHLH* cDNA and grown in the presence or absence of doxycycline (DOX). In (**f**) mRNA levels were normalized to *ACTB* and then to the no DOX *ACTB*-normalized value for that mRNA (n = 3). (**g**) Immunoblot analysis of representative OSRC-2 tumor lysates at study endpoint in Fig. 4j. (**h**, **i**) Individual body weight (BW) trajectories (**h**) and group mean ± SEM for BW change over time (**i**) of mice treated with vehicle, PT2399, or Zoledronic acid (ZOL). (**j**) Percent change in serum calcium levels from baseline to endpoint in each treatment group vehicle, PT2399, or ZOL. NTB = non–tumor-bearing controls (exact p values are indicated on the figure panels). (**k**) Tumor weights at endpoint for mice treated with vehicle, PT2399, or ZOL. (**l**–**n**) Early timepoint measurements of serum calcium from individual ZOL- and vehicle-treated mice that required early euthanasia due to cachexia. Data are presented as mean ± SEM. Statistical significance was assessed using unpaired two-tailed t-tests and two-way ANOVA test. Source data

We next performed loss-of-function and gain-of-function *PTHLH* studies to ask whether PTHrP was necessary or sufficient, respectively, for the rapid induction of cachexia by OSRC-2 cells. For the former, we performed orthotopic tumor assays with polyclonal OSRC-2 cells stably infected with a lentivirus expressing: (1) Cas9, (2) firefly luciferase and (3) either a *PTHLH* single guide RNA or a control (AAVS1) sgRNA. Tumor burden was monitored by serial bioluminescent imaging. CRISPR-mediated knockout (CRISPR KO) of *PTHLH* in OSRC-2 cells was verified by amplicon sequencing and dramatically decreased PTHrP secretion in vitro (Fig. 4a and Extended Data Fig. 4a). *PTHLH* mRNA levels, but not other HIF2-responsive mRNA levels, were modestly reduced in the CRISPR *PTHLH* KO cells, presumably because of mRNA destabilization resulting from indel mutations (Extended Data Fig. 4b–d).

Mice implanted with OSCR2 cells edited with the AAVS1 sgRNA developed cachexia and were euthanized when moribund. By contrast, the mice implanted with the OSRC-2 cells that had undergone CRISPR KO of *PTHLH* did not lose weight and were not killed until the last control mice needed to be euthanized (day 57) (Fig. 4b–d). The sg*PTHLH* tumors were significantly larger than the sgAAVS tumors (Fig. 4e), arguing that the absence of cachexia with the former was not because they were insufficiently large. Note that the sgAAVS tumors had to be excised earlier because of cachexia and hence this experiment does not indicate that *PTHLH* is a ccRCC suppressor. Serum PTHrP was below the sensitivity limits of the commercial ELISA kits we tested. However, PTHrP has also been implicated in humoral hypercalcemia^35^. Serum calcium levels were increased in mice with sgAAVS1 tumors compared to the mice with sg*PTHLH* tumors and to NTB, suggesting that PTHrP was elevated in mice with sgAAVS1 tumors (Fig. 4f).

To ask whether the *PTHLH* KO phenotype was on-target, we superinfected the *PTHLH* KO OSRC-2 cells with a lentivirus expressing a doxycycline (DOX), sgRNA-resistant, *PTHLH* complementary DNA that encoded PTHrP with a C-terminal V5 epitope tag (Fig. 4g and Extended Data Fig. 4e). We confirmed that inducible *PTHLH* expression did not affect other canonical HIF2-responsive mRNAs (Extended Data Fig. 4e,f). Mice orthotopically implanted with these cells and continuously fed DOX-containing chow, but not normal chow, developed hypercalcemia and cachexia about 4 weeks later (Fig. 4h–j and Extended Data Fig. 4g). Notably, removing DOX in the former mice, thus silencing *PTHLH*, normalized serum calcium, reversed the cachexia and prolonged survival (Fig. 4h–j and Extended Data Fig. 4g). These effects were again not explained by differences in tumor burden measured at the study endpoint (Fig. 4k). To determine whether hypercalcemia was the underlying cause for the cachectic phenotype, we treated OSRC-2 tumor-bearing mice with the calcium-lowering bisphosphonate zoledronic acid^36^ (ZOL; 120 µg kg^−1^ intraperitoneally every other day), with the HIF2 inhibitor PT2399 (45 mg kg^−1^ daily oral gavage) or with vehicle (Extended Data Fig. 4h–k). As before, vehicle-treated mice developed progressive weight loss and hypercalcemia, whereas PT2399 reversed both phenotypes independently of tumor weight. ZOL rapidly normalized serum calcium within 3–4 days of treatment initiation (Extended Data Fig. 4l–n), but did not reverse the cachexia. As a result, ZOL-treated mice ultimately succumbed to severe cachexia despite correction of hypercalcemia. These findings indicate that hypercalcemia is not necessary for the cachexia we observe in this model.

### Increased PTHrP expression is sufficient to induce cachexia

**Fig. 5:**
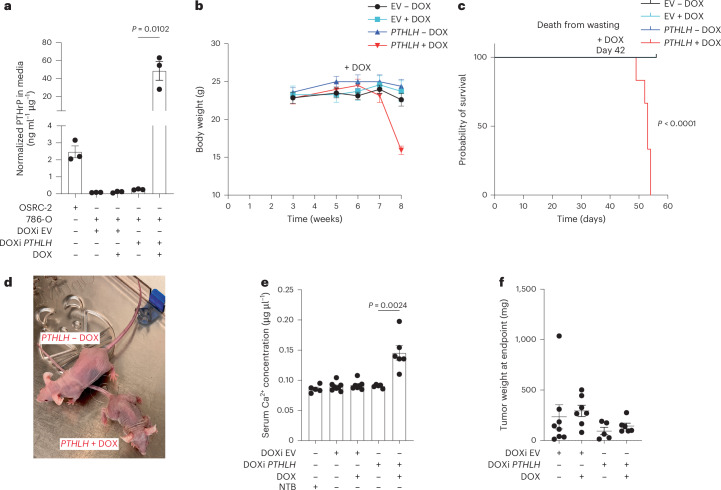
*PTHLH* is sufficient for the induction of cachexia by 786-O xenografts. **a**, PTHrP levels in conditioned media from 786-O cells infected with DOXi *PTHLH* lentivirus or corresponding DOXi EV and grown in the presence or absence of DOX, normalized to total cellular protein (*n* = 3, *P* = 0.0102). **b**,**c**, Body weights (**b**) and Kaplan–Meier survival curve (*P* < 0.0001) (**c**) for mice with xenografts formed by 786-O cells from **a** and maintained on chow with or without DOX. Group sizes: EV − DOX (*n* = 8), EV + DOX (*n* = 7), DOXi *PTHLH* − DOX (*n* = 5), DOXi *PTHLH* + DOX (*n* = 6). Statistical analysis was performed using the log-rank test. **d**–**f**, Representative mouse images (**d**), serum calcium levels (*P* = 0.0024) (**e**) and tumor weights (**f**) at study endpoint of mice from **b** and **c**. Each dot represents one independent mouse. Statistical significance was assessed using unpaired two-tailed *t*-tests.

**Extended Data Fig. 5:**
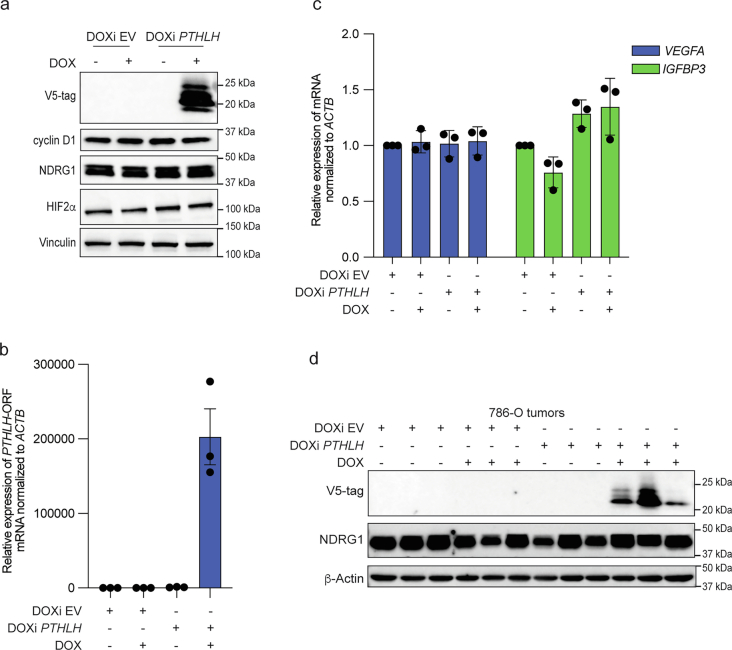
Extended analysis of *PTHLH* sufficiency for the induction of cachexia in 786-O xenografts. (**a**–**c**) Immunoblot (**a**) and qRT-PCR (**c**) analysis of 786-O cells infected with a doxycycline (DOX)-inducible *PTHLH* lentivirus or empty vector (EV). The *PTHLH* cDNA also encoded a 3’ V5 epitope tag. Cells were grown in the presence of 1 μg/mL DOX for 3 days where indicated. In (**b**) qRT-PCR was performed with a primer targeting the V5 tag nucleotide sequence and mRNA values were normalized to *ACTB* then to the EV, no DOX, *ACTB*-normalized mRNA value (n = 3). In (**c**) mRNA expression of *VEGFA* and *IGFBP3* was normalized to *ACTB* and then to the EV, no DOX, value for the corresponding mRNA (n = 3). (**d**) Immunoblot analysis of representative 786-O tumor lysates at study endpoint in Fig. 5e. In (**b**, **c**) data are presented as mean ± SEM. Source data

To address sufficiency, we infected 786-O with a lentivirus expressing a DOX-inducible *PTHLH* (DOXi *PTHLH*) cDNA that also encodes a 3′ V5 epitope tag or, as a control, the empty vector (DOXi EV). Treatment of the DOXi *PTHLH* cells with DOX increased PTHrP secretion in vitro, as expected, again without affecting other HIF2 target genes (Fig. 5a and Extended Data Fig. 5a–c). Next the DOXi *PTHLH* cells and DOXi EV cells were orthotopically implanted in nude mice fed normal chow. Tumor formation was monitored by serial bioluminescent imaging. Six weeks after implantation, the tumor-bearing mice were randomized to DOX-containing chow or normal chow. Mice with DOXi *PTHLH* tumors, but not DOXi EV tumors, rapidly developed cachexia and hypercalcemia, requiring euthanasia within several weeks, if fed the DOX-containing chow (Fig. 5b–e and Extended Data Fig. 5d). The tumors removed from these euthanized mice were not larger than tumors removed from the EV and no DOX control groups at the time the last DOXi *PTHLH* tumor-bearing mice needed to be euthanized (day 56) (Fig. 5f).

### HIF2–PTHrP signaling drives cachexia and hypercalcemia across preclinical models and patients

**Extended Data Fig. 6:**
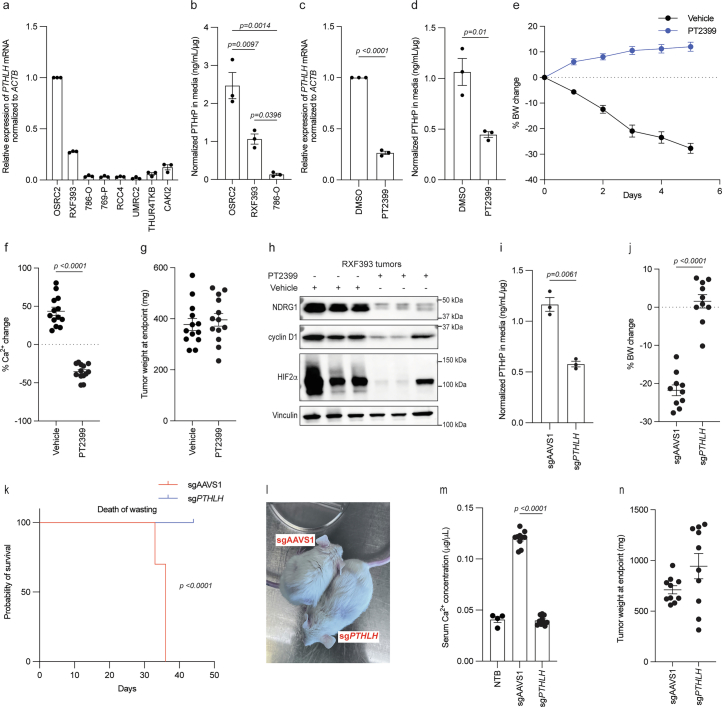
RXF393 induces cachexia via HIF2 and PTHrP. (**a**) *PTHLH* mRNA qRT-PCR across ccRCC cell lines. *PTHLH* mRNA levels were normalized to *ACTB* and then to the OSRC-2 cell *ACTB*-normalized value (n = 3). (**b**) PTHrP levels in media conditioned by OSRC-2, RXF393, or 786-O cells, normalized to total cellular protein (n = 3, exact p values are indicated on the figure panels). (**c**, **d**) *PTHLH* mRNA qRT-PCR (*p* < *0.0001*) (**c**) and PTHrP ELISA (*p* = *0.01*) (**d**) in OSRC-2 cells treated with 2 μM PT2399 or DMSO for 48 h. In (**c**) *PTHLH* mRNA levels were normalized to *ACTB* and then to the DMSO *ACTB*-normalized value (n = 3). In (**b**) PTHrP levels in the media was normalized to total cellular protein (n = 3). (**e**) Body weight trajectories of mice bearing RXF393 tumors treated with PT2399 (n = 13) or vehicle (n = 13). (**f**–**h**) percent change in serum calcium levels relative to baseline pre-treatment levels (*p* < *0.0001*) (**f**), tumor weight at the study endpoint (**g**), immunoblot analysis of representative RXF393 tumor lysates at study endpoint in (**h**) of mice bearing RXF393 tumors treated with PT2399 or vehicle as in **c**). (**i**) PTHrP ELISA of 2 days conditioned media from RXF393 cells infected to express sg*PTHLH* or sgAAVS1, normalized to total cellular protein (n = 3, *p* = *0.0061*). (**j**, **k**) Body weight change (*p* < *0.0001*) (**j**) and Kaplan-Meier survival curves (*p* < *0.0001*) (**k**) for mice bearing RXF393 tumors infected to express sg*PTHLH* (blue, n = 10) or sgAAVS1 (red, n = 10). (**i**–**n**) Representative mouse images (**i**), serum calcium levels (**m**) and tumor weight at study endpoint (**n**) of mice from (**j**). Statistical analysis for Kaplan-Meier was performed using the log-rank test. Data are presented as mean ± SEM. Statistical significance was assessed using unpaired two-tailed t-tests or two-way ANOVA. Source data

To ask about the generalizability of our preclinical findings, we surveyed *PTHLH* expression in seven additional ccRCC cell lines. RFX393 cells had the second highest *PTHLH* mRNA levels (after OSRC-2 cells) and secreted measurable amounts of PTHrP (Extended Data Fig. 6a,b), both of which were suppressed by PT2399 (Extended Data Fig. 6c,d). As was true for OSRC-2 cells, RXF393 cells induced cachexia and hypercalcemia in subcutaneous xenograft assays and these two phenotypes were reversed by PT2399 (Extended Data Fig. 6e–h) and prevented by CRISPR KO of *PTHLH* without commensurate changes in tumor growth (Extended Data Fig. 6i–n). Therefore, upregulation of PTHrP by HIF2 causes cachexia and hypercalcemia in both the OSRC-2 and RXF393 models.

**Extended Data Fig. 7:**
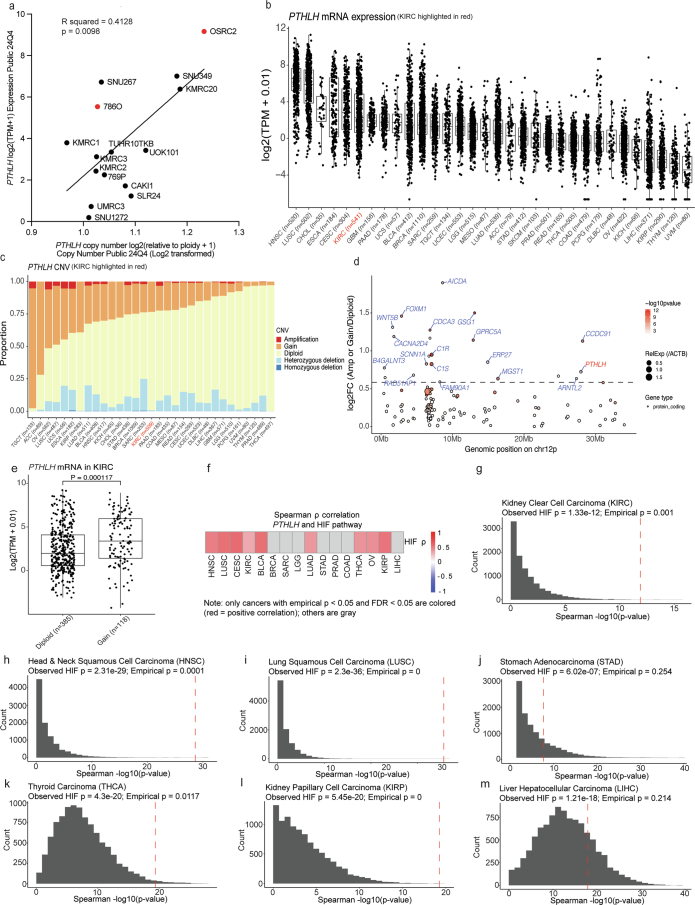
*PTHLH* is increased in KIRC, associated with copy number variation and gene expression in OSRC-2 and TCGA analysis, and correlates with a HIF transcriptional signature across multiple TCGA tumor types. (**a**) Scatter plot showing **the correlation between** ***PTHLH*** **gene copy number and mRNA expression in ccRCC cell lines**. The x-axis shows ***PTHLH*** copy number (log2 relative to ploidy +1), and the y-axis shows ***PTHLH*** mRNA expression (log2 TPM + 1). A linear regression curve was fitted using GraphPad Prism (two-sided F-test for slope: F(1, 13) = 9.14, P = 0.0098, R^2^ = 0.4128). OSRC-2 and 786-O are red labeled. Data extracted from Depmap.com 24Q4 dataset in Feb/09/2025. (**b**) Box plot of *PTHLH* mRNA expression across TCGA tumor types (log2 TPM + 0.01), sample numbers per tumor type are indicated on the x-axis (each dot = one tumor), KIRC (Kidney Renal Clear Cell Carcinoma) is highlighted in red font. (**c**) Proportional bar plot showing *PTHLH* copy number variation (CNV) across TCGA tumor types. Sample numbers per tumor type are indicated on the x-axis (each dot = one tumor), KIRC is highlighted in red font. (**d**) Scatter plot log2 fold change of mRNA expression of copy-gain/ copy-neutral samples for genes on chromosome 12p in TCGA KIRC samples, with *PTHLH* highlighted as a significantly amplified gene. Circle size represents relative expression (normalized to *ACTB*), and color intensity indicates -log10 p-value significance (*P* values were calculated using a two-sided unpaired Wilcoxon rank sum test). The dashed line indicates fold change = 1.5. (**e**) Box plot showing *PTHLH* mRNA expression in TCGA KIRC samples with diploid (n = 385) or gain (n = 118) copy number states. Statistical significance (P = 0.000117) was assessed using two-sided unpaired Wilcoxon rank sum test. (**f**) Heatmap showing Spearman correlation coefficients (ρ) between *PTHLH* expression and the HIF transcriptional signature (*ADM*, *IRS2*, *EGLN3*, *EDN1*, *VEGFA*, *SLC2A1*, *TGFA*, *APOL1*, *CCND1*, *STC1*, *NDRG1*) across multiple TCGA cancer cohorts. Statistical significance was assessed using Pearson’s correlation (two-sided). Only associations meeting both statistical significance (FDR < 0.05) and empiric significance (empirical p-value < 0.05) are colored (red = positive correlation); non-significant associations are grey. (**g**–**m**) Examples of empiric p-value distributions for selected cancer types (no error bars are included). For each cancer type, the observed correlation p-value between *PTHLH* and the HIF signature (red dashed line) was compared to the distribution of correlation p-values obtained from 10,000 random gene sets of identical size to the HIF signature. Empirical p values were calculated using these distributions and are one-sided. This approach addresses potential false positives and confirms that several cancer types including HNSC show robust, highly significant associations. For box plots (**b**, **e**), the center line represents the median, box bounds represent the interquartile range (IQR), whiskers extend to 1.5 x IQR, and each dot represents an individual TCGA tumor sample.

*PTHLH* resides on chromosome 12p in a region that is amplified in different histologic types of kidney cancer, including ccRCC^37^, as well as in other cancers, such as pancreatic ductal adenocarcinoma^38^. Notably, an analysis of available ccRCC cell lines from DepMap^39^ revealed that the copy number of this region is increased in OSRC-2 cells compared to 786-O cells and that *PTHLH* expression positively correlates with *PTHLH* copy number across ccRCC cell lines (Extended Data Fig. 7a). Using TCGA data, we confirmed that *PTHLH* is frequently highly expressed in human ccRCC, especially in those who have gained or amplified chromosome 12p (Extended Data Fig. 7b–e). We performed a comprehensive pan-cancer transcriptomic analysis and found that *PTHLH* expression strongly correlates with a HIF transcriptional signature across multiple tumor types, with particularly strong associations in head and neck squamous cell carcinoma (HNSC) and lung squamous cell carcinoma (Extended Data Fig. 7f–m). Moreover, TCGA analysis revealed that HNSC exhibits the highest overall *PTHLH* mRNA expression levels among the tumor types we examined (Extended Data Fig. 7b).

To begin to address the clinical utility of our findings, we studied patients with ccRCC treated with the allosteric HIF2 inhibitor belzutifan for whom appropriate plasma samples and clinical information were available. Comparable patients treated with an immune checkpoint inhibitor (ICI), or a VEGF receptor tyrosine kinase inhibitor (TKI) served as controls, with the caveat that none of the patients treated with belzutifan received it as their first line of therapy, in contrast to most of the patients treated with ICI or TKI, in whom the ICIs and TKIs were frequently used as frontline therapy (Extended Data Table 1). Plasma PTHrP was measured in a Mayo Clinic CLIA Laboratory^40^.

**Fig. 6:**
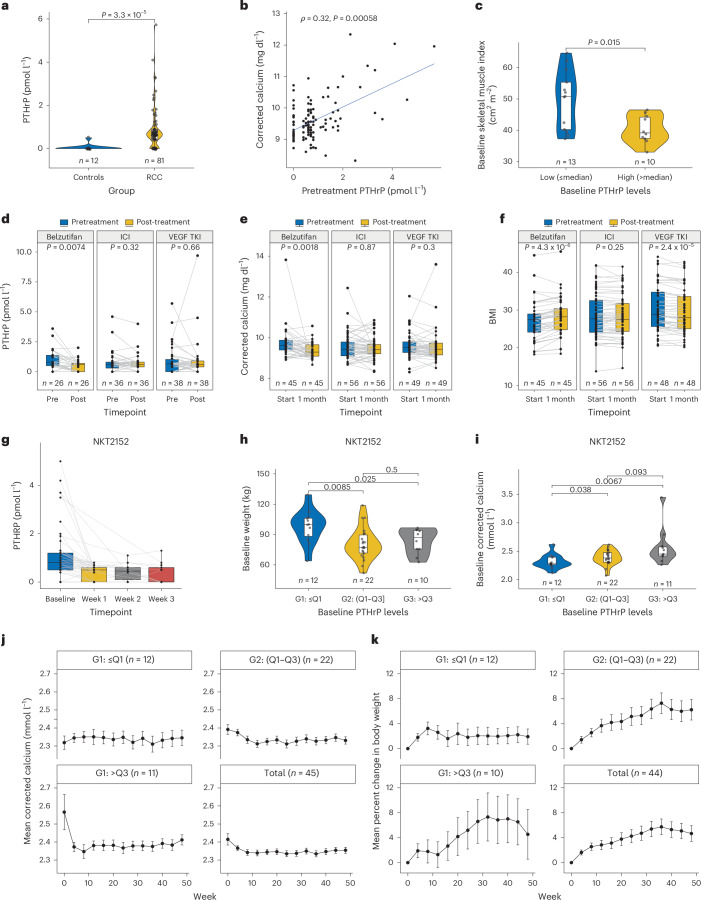
Treatment of kidney cancer hypercalcemia and cachexia with HIF2 inhibitors. **a**, Plasma PTHrP levels in untreated healthy controls (*n* = 12) and patients with RCC (*n* = 81). **b**, Correlation between pretreatment plasma PTHrP levels and pretreatment albumin-corrected calcium levels (*n* = 112). **c**, Skeletal muscle index (cm^2^ m^−^^2^) at baseline in patients stratified by circulating PTHrP levels (*n* = 23). Patients were divided into low (≤median, 0.8 pmol ml^−1^; *n* = 13) and high (>median; *n* = 10) PTHrP groups. **d**–**f**, Plasma PTHrP levels (**d**), corrected serum calcium levels (**e**) and body mass index (BMI) (**f**) in patients treated with belzutifan (*n* = 26 (**b**), *n* = 45 (**c**), *n* = 43 (**d**)), ICIs (*n* = 36 (**b**), *n* = 56 (**c**), *n* = 56 (**d**)) or VEGF TKIs (*n* = 38 (**b**), *n* = 49 (**c**), *n* = 48 (**d**)), pre- and post-treatment. **g**, Plasma PTHrP levels over time (baseline, week 1, week 2 and week 3) in patients with RCC treated with the HIF2 inhibitor NKT2152 (*n* = 45). **h**, Baseline body weight of patients with RCC stratified by plasma PTHrP levels: low (<quartile 1 (Q1), *n* = 12), mid (Q1–Q3, *n* = 22) and high (>Q3, *n* = 10). **i**, Baseline albumin-corrected calcium levels stratified by baseline plasma PTHrP levels: low (<Q1, *n* = 12), mid (Q1–Q3, *n* = 22) and high (>Q3, *n* = 11). **j**,**k**, Mean corrected serum calcium (**j**) and mean percent change in body weight (**k**) over 50 weeks in patients with RCC from **h** treated with NKT2152, stratified by plasma PTHrP levels. Windowing algorithm and Last Observation Carried Forward were applied for plotting **j** and **k**. Data in **j** and **k** are shown for patients with low (<Q1, *n* = 12), mid (Q1–Q3, *n* = 22) and high (>Q3, *n* = 11 (**j**), *n* = 10 (**k**), due to one subject missing baseline body weight information) baseline PTHrP levels. For box and violin plots (**c**–**i**), the center line represents the median, the box bounds represent the interquartile range, whiskers extend to 1.5× interquartile range, and each dot represents one patient. Data in **j** and **k** are presented as mean ± s.e.m. Statistical significance was assessed for **a**, **c**, **h** and **i** by using two-sided unpaired Wilcoxon rank sum test, for **d**–**f** using the two-sided paired Wilcoxon signed-rank test, and for **b** using two-sided Spearman’s rank correlation coefficient (*ρ*). G1, group 1; G2, group 2; G3, group 3.

**Extended Data Fig. 8:**
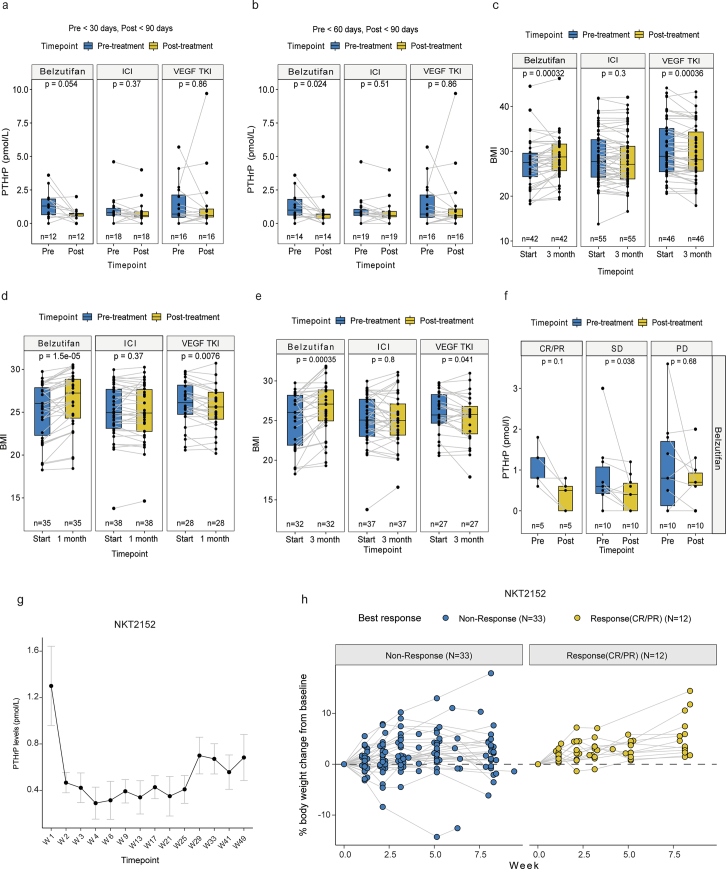
Additional analysis of hypercalcemia and cachexia treatments in RCC patients using HIF2 inhibitors, ICIs, and VEGF TKIs. (**a** and **b**) Plasma PTHrP levels in ccRCC patients treated with belzutifan (**b**: n = 12, **c**: n = 14), immune checkpoint inhibitors (ICIs; **b**: n = 18, **c**: n = 19), or VEGF tyrosine kinase inhibitors (TKIs; **b**: n = 16, **b**: c = 16) pre-treatment and post-treatment, stratified by different pre- and post-treatment time windows. (**c**) BMI in ccRCC patients treated with belzutifan (n = 42), ICIs (n = 55), or VEGF TKIs (n = 46), measured pre-treatment and after 3 months. (**d**, **e**) Changes in BMI for patients with pre-treatment BMIs <30 after being treated for 1 month (**d**) or 3 months (**e**) with belzutifan, ICI, or VEGF TKIs. (**f**) Plasma PTHrP levels in ccRCC patients treated with belzutifan, grouped by response category: CR/PR (complete/partial response, n = 5), SD (stable disease, n = 10), and PD (progressive disease, n = 10), measured pre-treatment and post-treatment. (**g**) Plasma PTHrP levels over time (up to Week 49) in ccRCC patients treated with NKT2152 (n = 14). Error bars represent standard error (SE). (**h**) Longitudinal changes in body weight (%) from baseline in patients treated with NKT2152, stratified by response status. Non-responders (n = 33; stable disease or progressive disease, blue) and responders (n = 12; partial or complete response, yellow). Data in (**g**, **h**) are presented as mean ± SEM. For box plots (**a**–**f**), the center line represents the median, the box bounds represent the interquartile range (IQR), whiskers extend to 1.5 x IQR, and each dot represents one patient. Statistical analysis and significance were assessed for (**a**–**f**) using the two-sided paired Wilcoxon signed-rank test.

We identified 26 patients with plasma samples before and after belzutifan, 36 patients with plasma samples before and after ICI therapy and 38 patients with samples before and after TKI therapy. We confirmed that plasma PTHrP was elevated in the patients with ccRCC compared with 12 age-matched healthy volunteers (Fig. 6a). Pretreatment PTHrP levels positively correlated with the pretreatment corrected calcium levels (Fig. 6b). In this cohort, patients with baseline PTHrP levels above the median had significantly lower baseline skeletal muscle index compared to those with lower PTHrP levels (Fig. 6c). Belzutifan, but not ICI or VEGF TKI, suppressed plasma PTHrP (Fig. 6d and Extended Data Fig. 8a,b) and corrected calcium after 1 month of treatment (Fig. 6e). Notably, the patients treated with belzutifan gained weight during this period, whereas weight was unchanged in the ICI group and fell in the VEGF TKI group (Fig. 6f and Extended Data Fig. 8c). The same pattern emerged when this analysis was restricted to patients with pretreatment body mass index <30 (Extended Data Fig. 8d,e). These effects of belzutifan did not appear to be a secondary consequence of tumor shrinkage and loss of viable tumor cells because (1) they occurred rapidly, (2) were observed in both belzutifan responders and nonresponders, and (3) the clinical response rate to belzutifan, if anything, trended lower than the response rates to ICI and VEGF TKI in our cohorts (Extended Data Fig. 8f and Extended Data Table 1).

To further probe the robustness of these findings, we obtained plasma samples and clinical data for 45 patients with ccRCC who were treated with the allosteric HIF2 inhibitor NKT2152, which is currently under clinical development^41^. NKT2152 caused a rapid and sustained suppression of PTHrP (Fig. 6g and Extended Data Fig. 8g). We then subdivided the patients based on their pretreatment PTHrP values as follows: group 1, the 12 patients with the lowest PTHrP levels; group 2, the 22 patients with baseline PTHrP levels in the 25th to 75th percentile of PTHrP levels; and group 3, the 11 patients with the highest PTHrP levels. A higher proportion of group 3 patients had stage IV disease, presumably reflecting the anticipated correlation between PTHrP levels and total tumor burden (Extended Data Table 2). It is also known that high *PTHLH* levels correlate with worse outcomes in ccRCC^42^. As expected from our preclinical findings, group 3 had the lowest body weights and highest corrected serum calcium levels of the three groups at baseline (Fig. 6h,i) and appeared to derive the greatest benefit from NKT2152 treatment with respect to these two measures (Fig. 6j,k). Note that one group 3 patient was omitted from Fig. 6h,k because they did not have a baseline body weight value. By contrast, the clinical response rates did not appear to be higher in group 3 than in the other two groups (Extended Data Table 2). As was true for belzutifan, weight gain in patients treated with NKT2152 was observed even in patients with stable or progressive disease, indicating that cachexia reversal can occur independently of tumor shrinkage (Extended Data Fig. 8h). Sex-disaggregated analyses for patients treated with belzutifan (Extended Data Table 1) and those treated with NKT2152 (Extended Data Table 2), based on sex assigned at birth, are provided in the source data files and showed that key trends were similar in males and females. Therefore, two different allosteric HIF2 inhibitors appear to mitigate two of the common paraneoplastic syndromes associated with ccRCC.

## Discussion

Paraneoplastic syndromes are common in patients with ccRCC and often complicate their management^12–14^. In this study, we strengthened the earlier conclusion that *PTHLH* is a direct HIF2 target gene^24,25,32,33^, although our findings do not exclude the possibility that pVHL loss also stabilizes the *PTHLH* mRNA, as suggested by others previously^43^. We provide further evidence that the *PTHLH* gene product, PTHrP, causes the hypercalcemia that frequently complicates ccRCC^42,44–46^. More importantly, we provide preclinical and clinical evidence that PTHrP also causes cachexia that frequently complicates ccRCC and that both hypercalcemia and cachexia in ccRCC can be managed with pharmacological HIF2 inhibitors. Notably, not all patients with ccRCC develop hypercalcemia and cachexia^14^. The development of these two ccRCC complications is likely to be influenced by multiple factors, including the degree of HIF dysregulation, *PTHLH* copy number variation and the epigenetic accessibility of *cis*-regulatory HIF-binding elements (for example, whether they are unmethylated). Although hypercalcemia can, itself, cause nausea, vomiting and decreased appetite, the cachexia in our mouse models was not associated with decreased caloric intake. It was instead associated with adipocyte changes previously shown to be mediated by the PTHrP receptor^18,19^.

PTHrP facilitates cachectic wasting through the engagement with its cognate receptor, parathyroid hormone 1 receptor, on adipocytes^18,19,47,48^. Consistent with previous results, we found that PTHrP leads to adipose tissue wasting and causes a switch from an energy-storing phenotype to an energy-consuming phenotype in adipocytes, as marked by Ucp1 induction. Notably, the role of Ucp1-mediated browning in driving cachectic wasting remains controversial. To test the functional role of Ucp1 in wasting, whole-body *Ucp1* deletion animals (Ucp1^−/−^) have been used, which demonstrated that Ucp1 loss blocks cachectic wasting in some contexts^49^, but not others^50^. Thus, Ucp1-independent mechanisms, such as increased lipolysis or decreased lipogenesis, have been suggested to also contribute to cachexia. Although we noted increased Ucp1 expression in cachectic adipose, we did not test its functional role in driving wasting, and it remains to be explored whether lipolytic and lipogenic pathways also contribute to PTHrP-mediated wasting in this context. Tumor-derived PTHrP has recently been shown to downregulate de novo lipogenesis in adipose tissue from mouse models of pancreatic cancer-associated cachexia^48^. Furthermore, patients with high circulating PTHrP demonstrate increased weight loss and whole-body fat oxidation independent of peripheral lipolysis^23^. The interplay between browning, lipogenesis and lipolysis during cachexia is complex, and the role of PTHrP in regulating these processes in RCC-mediated wasting warrants further study. Notably, cachexia occurred rapidly in the OSRC-2 model of ccRCC cachexia, which necessitated early euthanasia of the tumor-bearing mice. At the early time points we were able to study in this model, adipose tissue wasting predominated, suggesting that this model reflects an early stage of cachexia. Nonetheless, ANCOVA adjusting for pretreatment body weight revealed higher gastrocnemius mass in PT2399-treated mice compared to vehicle, whereas quadriceps showed a treatment-by-body weight interaction that precluded a definitive conclusion. Thus, skeletal muscle involvement is detectable but modest at these time points and is mitigated by HIF2 inhibition, while adipose tissue remains the principal early target. Supporting the clinical relevance of this observation, patients with baseline PTHrP levels above the median had significantly lower skeletal muscle index at baseline than those with lower PTHrP levels, consistent with a link between elevated PTHrP and reduced muscle mass^19^. As such, PTHrP-targeted interventions, either directly through anti-PTHrP therapies^38,51,52^ or indirectly by targeting the HIF2–PTHrP axis, might serve to block the onset of cachexia.

*PTHLH* and PTHrP are downregulated acutely with HIF2 inhibitors and thus can be viewed as pharmacodynamic markers. Downregulation of HIF2 activity is presumably necessary, although not necessarily sufficient, for the antitumor effects of HIF2 inhibitors in ccRCC, as evidenced by the OSRC-2 model. Moving forward it will be important, as larger clinical datasets with appropriate biospecimens become available, to ask whether elevated baseline PTHrP values and/or acute downregulation of PTHrP after treatment are positive predictive biomarkers for achieving disease control, including RECIST (response evaluation criteria in solid tumors) responses, with HIF2 inhibitors. Earlier work implicated PTHrP as a therapeutic target in ccRCC^53^, although in our model, genetic ablation of *PTHLH* did not impede tumor growth. A caveat, however, is that these earlier studies used neutralizing antibodies and antagonist peptides that would affect both tumor-derived and host-derived PTHrP, in contrast to our studies^38,53–56^.

In our preclinical models, reversal of cachexia with HIF2 inhibition occurred rapidly and could be dissociated from changes in tumor mass. We found that patients treated with HIF2 inhibitors tended to gain weight on therapy, in contrast to patients treated with standard of care agents such as ICIs or VEGF receptor TKIs. These findings are consistent with suppression of PTHrP reversing both clinically occult and clinically overt cachexia physiology. A caveat, however, is that our clinical data with respect to weight loss across treatment groups could be confounded by therapy-induced toxicities caused by TKIs and ICIs, as well as by differences in patient characteristics across the different treatment groups, each of which was comprised of a relatively small number of patients for whom we had access to appropriate samples. Clearly it will be important to confirm our findings in larger studies. It will also be of interest to determine whether concurrent treatment with belzutifan mitigates the risk of weight loss in patients treated with other ccRCC therapies, including VEGF TKIs.

The BirA–ER system, combined with quantitative LC–MS/MS, appears to be a robust engine for the discovery of proteins that are differentially secreted by cells under different conditions, such as, in our case, the presence or absence of a HIF2 inhibitor. We discovered multiple known HIF-responsive growth factors, including IGFBP3, PTHrP and VEGF-A^57^. It is likely that HIF2-dependent secreted proteins have roles in some of the other kidney cancer paraneoplastic syndromes for which the mechanism remains unknown. Although we focused on secreted proteins that were repressed by PT2399, secreted proteins that are induced after HIF2 blockade could also provide insights into HIF2 biology and lend themselves to serve as pharmacodynamic markers that would rise (‘up assay’), rather than fall, in serum when HIF2 is successfully inhibited.

It will be important to determine whether intratumoral hypoxia and HIF induce PTHrP in other cancers, such as pancreatic cancers^23^, head and neck cancers^58^, lung cancer^18^ and colorectal cancer^59^, where PTHrP has been implicated in cachexia and, if so, to determine which HIF paralog is responsible. In this regard, our pan-cancer analyses suggest that *PTHLH* is significantly correlated with HIF transcriptional activity in multiple cancer types, including HNSC and lung squamous cell carcinoma. Moreover, our HNSC cell line data suggest that both HIF1α and HIF2 drive *PTHLH* expression in this setting. Notably, the drug-binding pocket for HIF2α is much smaller than the corresponding pocket in HIF1α, which has thus far precluded the development of HIF1α inhibitors analogous to belzutifan and NKT2152^60^. Nonetheless, a number of drugs that directly or indirectly inhibit HIF1α have been described^61^.

Our findings highlight an important role for PTHrP in ccRCC cachexia. In other settings, GDF15 contributes to cancer cachexia, and it is conceivable that both secreted factors are operative in some settings. An anti-GDF15 monoclonal antibody, ponsegromab, was recently shown to ameliorate cachexia caused by nonsmall cell lung cancer and pancreatic cancer^62^, and CLIA-certified assays exist for measuring PTHrP and GDF15 in plasma. We envision the use of such assays in the future to tailor the use of PTHrP antagonists (direct or indirect via HIF) and GDF15 antagonists to treat cachexia.

## Methods

### Cell lines

OSRC-2 cells (males) were obtained from the Riken Cell Bank (RCB0735) and RXF393 cells (males) were obtained from the National Cancer Institute (DCTD Tumor Repository). Both cell lines were maintained in RPMI 1640 (GIBCO, cat. no. 11875093) supplemented with 10% FBS (GeminiBio, cat. no. 100-106), 100 U ml^−1^ penicillin and 100 μg ml^−1^ streptomycin (P–S; Gibco, cat. no. 25200056). 786-O cells (American Type Culture Collection (ATCC) CRL-1932, males) and 293T cells (ATCC CRL-3216, females) were obtained from the Kaelin Laboratory stocks and were maintained in Dulbecco’s modified Eagle’s medium (DMEM; GIBCO, cat. no. 11965092) supplemented with 10% FBS and P–S as above. SCC-9 (ATCC CRL-1629, males) and SCC-4 (ATCC CRL-1624, males) cells were obtained from ATCC and were maintained in DMEM–F-12, GlutaMAX (GIBCO, cat. no. 10565018) supplemented with 400 ng ml^−1^ hydrocortisone (Sigma-Aldrich, cat. no. H0135-1MG) and 10% FBS and P–S as above. Lentiviral infected cells were maintained as polyclonal cells under drug selection. OSRC-2 and 786-O cells were selected with 800 μg ml^−1^ G418, 10 μg ml^−1^ blasticidin or 2 μg ml^−1^ puromycin. RXF393 cells were selected with 400 μg ml^−1^ G418 or 2 μg ml^−1^ puromycin. All cell lines were confirmed to be mycoplasma-free using a MycoAlert Mycoplasma Detection kit (Lonza, cat. no. LT07-318). The OSRC-2, RXF393, 786-O, 293T, SCC-9 and SCC-4 cell lines used in this study were authenticated by short tandem repeat profiling. Authentication was performed by the Cell Line Authentication Service at the ATCC in 2024 and 2025. None of the cell lines used in this study are listed as misidentified or cross-contaminated in the ICLAC database.

### Plasmids

The sgRNA-resistant *PTHLH* cDNA was created by site-directed mutagenesis using an Agilent QuikChange II XL Site-Directed Mutagenesis Kit (Agilent, cat. no. 200522) according to the manufacturer’s instructions, on a pENTR221 plasmid containing a wild-type *PTHLH* cDNA (Horizon Discovery, cat. no. OHS5894-202497874). The primers used for site-directed mutagenesis and subcloning are listed below. The wild-type and mutant *PTHLH* cDNAs from the respective pENTR221 plasmids were then shuttled into pLIX_403-Puro vector (Addgene, cat. no. 41395) or pLIX403-ccdB-Blast (Addgene, cat. no. 158560) using Gateway LR Clonase II Enzyme mix (Invitrogen, cat. no. 11791100) according to the manufacturer’s instructions. The lentiCRISPR v2-Puro (Addgene, cat. no. 52961), lentiCRISPR v2-Blast (Addgene, cat. no. 83480), psPAX2 (Addgene, cat. no. 12260) and pMD2.G (Addgene, cat. no. 12259) were obtained from Addgene. The pLL3.7_EF1a_Fluc-neo vector was obtained from the Kaelin Laboratory stocks. The pLX304-BirA-G3*-ER construct was generated by recombining the BirA-G3*-ER ORF cDNA from a pDONR gateway entry clone (a gift from N. Perrimon’s Laboratory, Harvard Medical School) into the pLX304 vector (Addgene, cat. no. 25890) using the Gateway cloning methodology as described above. The sgRNA expression vectors were digested with BsmBI (Thermo Fisher Scientific, cat. no. ER0451) and ligated to annealed oligonucleotides encoding the desired sgRNAs using T4 ligase (NEB, cat. no. M0202M) as described in ref. ^63^. All *PTHLH* cDNA and sgRNA inserts were validated by DNA Sanger sequencing.

### Targeted mutagenesis primers

*PTHLH* sg*PTHLH* Resistant Forward 5′-CAGATGGTGAAGGAAGAACCGTCGCCGCAAGTCCTGAATGGACTTCCCCTTGTCAT-3′

*PTHLH* sg*PTHLH* Resistant Reverse 5′- ATGACAAGGGGAAGTCCATTCAGGACTTGCGGCGACGGTTCTTCCTTCACCATCTG-3′

### sgRNA sequences (including BsmBI site)

sg*PTHLH* sense 5′-CACCGCATACGGGCAGCACGACGCG-3′

sg*PTHLH* antisense 5′-AAACCGCGTCGTGCTGCCCGTATG-3′

sg*HIF1A* sense 5’- CACCGTATGTGTGAATTACGTTGTG-3’

sg*HIF1A* antisense 5’-AAACCACAACGTAATTCACACATA-3’

sg*ARNT* sense 5’-CACCGTGGGGAACCTCACTTCGTGG-3’

sg*ARNT* antisense 5’-AAACCCACGAAGTGAGGTTCCCCA-3’

sg*EPAS1* sense 5′-CACCGTCATGAGGATGAAGTGCA-3′

sg*EPAS1* antisense 5′-AAACTGCACTTCATCCTCATGA-3′

### Chemicals

PT2399 (Merck & Co.) was reconstituted in sterile DMSO to achieve a stock concentration of 2 mM and added to media to achieve a final concentration of 2 μM. Biotin (Sigma-Aldrich, cat. no. B4501-1G) was prepared to a stock solution of 200 mM in sterile water and added to media to achieve the desired final concentration of 12.5 µM. DOX (Takara, cat. no. 631311) was reconstituted in sterile water to achieve a stock concentration of 1 mg ml^−1^ and added to media to achieve a final concentration of 1 μg ml^−1^. ZOL (Selleck Chemicals, cat. no. S1314-100 mg) was prepared as a stock solution of 1 mg ml^−1^ in PBS.

### Immunoblot analysis

Immunoblot analysis was performed as described elsewhere^64^. The primary antibodies used included: rabbit anti-cyclin D1 (Cell Signaling Technologies, cat. no. 2978), rabbit anti-HIF2α (D6T8V; Cell Signaling Technology, cat. no. 29973S), rabbit anti-NDRG1 (Cell Signaling Technology, cat. no. 5196S), mouse anti-vinculin (Sigma, cat. no. V9131), rabbit anti-V5-tag (Cell Signaling Technology, cat. no. 13202S), mouse anti-β-actin (Cell Signaling Technology, cat. no. 3700S), anti-streptavidin–HRP (Cell Signaling Technology, cat. no. 3999S), rabbit anti-IGFBP3 (Cell Signaling Technology, cat. no. 25864S) and rabbit anti-MMP2 (D2O4T; Cell Signaling Technology, cat. no. 87809S). Unless otherwise noted, antibodies were used at 1:1,000 in 5% BSA; anti-β-actin was used at 1:2,000.

### Lentivirus preparation and infection

Lentivirus preparation and infection were performed as described in ref. ^64^. Briefly, lentiviruses were prepared by seeding 1.5 × 10^6^ 293T cells in a 60-mm plate, followed the next day by transfection with 1 μg of lentiviral vector DNA, 0.75 μg of psPAX2 and 0.25 μg of pMD2.G in 250 μl of Opti-MEM with 6 μl of TransIT-VirusGEN reagent (Mirus, cat. no. MIR 6700). After a 15-min incubation at room temperature, the mixture was added dropwise to 293T cells in 3 ml of P–S-free DMEM with 10% FBS. The cells were incubated overnight, and then the medium was replaced with DMEM containing 30% FBS and 1% P–S. Virus-containing media were collected at 24 h and 48 h, pooled, centrifuged, filtered and stored in 1.5-ml aliquots at −80 °C. For infection, 2 × 10^6^ target cells were plated in six-well plates with 1 ml of lentivirus, 10 μg ml^−1^ polybrene (Santacruz, cat. no. sc-134220) and 1.5 ml of media per well. Plates were gently shaken, centrifuged at 200*g* for 30 min at 30 °C, then incubated overnight. The following day, cells were trypsinized, transferred to 10-cm plates, and cultured with the appropriate drug for selection of stable cell lines.

### BirA–ER labeling and streptavidin pulldown

For each condition to be tested, 4 × 10^6^ cells were seeded in 30 ml of culture medium in 15-cm dishes. The next day, the cells were rinsed three times with Dulbecco’s phosphate-buffered saline (DPBS; Gibco, cat. no. 14190144) to remove residual culture media. The cells were then treated with DMSO, 2 µM PT2399, DMSO + 12.5 µM biotin or PT2399 + 12.5 µM biotin in 18 ml of DMEM supplemented with 1% P–S and 0.5% FBS, in duplicate. Forty-eight hours later the media was collected, centrifuged at 50*g* for 10 min at 4 °C and filtered through a 0.45-µm filter to remove cellular debris. The media was concentrated using Amicon Ultra-15 concentrators (Millipore, cat. no. UFC900324) by two successive spins at 358*g* for 15 min at 4 °C in an Eppendorf 5810R-15 amp version tabletop centrifuge, each time discarding the flow-through. To fully remove residual biotin, 15 ml of DPBS was added to the concentrated media sample and centrifuged using the same centrifugation conditions, again discarding the flow-through. If more than 1 ml remained after the final spin, additional spins were conducted until the retained volume was reduced to ~1 ml, which was then transferred to a 1.5-ml Eppendorf tube.

In parallel, cell lysates were also prepared to assess intracellular biotin labeling and protein expression. After removing media for the pulldown experiments, cell monolayers were rinsed twice with DPBS and detached from the culture surface with a cell lifter in 1 ml of PBS. The cell suspension was then transferred to a 1.5-ml Eppendorf tube and pelleted in a benchtop centrifuge Eppendorf 5810R-15 amp version tabletop centrifuge at 50*g* for 5 min at 4 °C. DPBS was aspirated and the cell pellet was lysed in 100 µl of RIPA buffer (Life Technologies, cat. no. 89901) supplemented with protease (Roche, cat. no. 1836170) and phosphatase (Sigma-Aldrich, cat. no. 04906837001) inhibitors and rotated at 4 °C 30 min. Lysates were cleared of insoluble debris by centrifugation at 17,000*g* for 15 min at 4 °C in an Eppendorf microcentrifuge and stored at −80 °C or used immediately for western blot analysis.

To prepare streptavidin magnetic beads (Thermo Fisher Scientific, cat. no. 65002), the beads were first resuspended by thorough vortexing until no beads adhered to the bottom of the bottle when inverted. Then 500 µl of resuspended beads were transferred to a 15-ml conical tube, adjusted to 10 ml with Tris-buffered saline with 0.1% Tween 20, and spun at 358*g* in an Eppendorf 5810R-15 amp version tabletop centrifuge for 10 min at 4 °C. The supernatant was removed by aspiration with a 10-ml pipette and the beads were resuspended in 1 ml of RIPA buffer supplemented with protease and phosphatase inhibitors and transferred to 1.5-ml Eppendorf tubes. The tubes with the beads were transferred to a magnetic strip, washed twice with 1 ml of fresh RIPA and resuspended in 500 µl of Tris-buffered saline with 0.1% Tween 20. Beads were stored on ice until use.

For capture of biotinylated proteins, the volume of each concentrated media sample was brought up to ~1 ml with DPBS. Then 30 µl of resuspended streptavidin beads were added, ensuring thorough beads resuspension before addition. Samples were incubated overnight on an end-over-end rotator at 4 °C. The next day, the tubes were quick-spun in an Eppendorf microcentrifuge to collect any liquid from the caps and then transferred to a magnetic strip to pellet the beads. The supernatant was removed by aspiration with a P1000 pipette. The beads were washed twice with 1 ml of RIPA buffer containing protease and phosphatase inhibitors, followed by sequential washes with 1 ml of 1 M KCl, 0.1 M Na_2_CO_3_ followed by 2 M urea in 10 mM Tris–HCl, pH 8. The beads were then washed twice with RIPA, twice with DPBS and finally resuspended in 1 ml of DPBS. The urea washes were performed in batches of no more than four samples, immediately followed by an RIPA wash to preserve sample integrity. A 35-µl aliquot of each bead suspension was removed for quality control, and the remainder was stored at −80 °C for MS analysis. For quality control, the beads were pelleted by a quick spin in an Eppendorf microcentrifuge. The DPBS was carefully aspirated and the beads were resuspended in 30 µl of 1× Laemmli sample buffer with 7.5 mM biotin to facilitate protein elution. For quality control experiments the beads were boiled at 95 °C for 10 min, then either frozen at −80 °C or used immediately for western blot analysis. Otherwise the beads were used for on-bead digestion, as described below.

### On-bead trypsin digestion of biotinylated proteins

Peptides bound to streptavidin magnetic beads were washed four times with 200 µl of 50 mM Tris–HCl (pH 7.5) buffer. After removing the final wash, the beads were incubated twice at room temperature in 80 µl of the digestion buffer—2 M urea, 50 nM Tris–HCl, 1 mM dithiothreitol (DTT) and 0.4 µg trypsin—while shaking at 1,000 rpm. The first incubation lasted 1 h, followed by the second incubation of 30 min. After each incubation, the supernatant was collected and transferred to a separate tube. The beads were then washed twice with 60 µl of 2 M urea and 50 mM Tris–HCl buffer. The resulting washes were combined with the digestion supernatant. The pooled eluate of each sample was then spun down at 5,000*g* for 30 s to collect the supernatant. The samples were subsequently reduced with 4 mM DTT for 30 min at room temperature with shaking at 1,000 rpm, followed by alkylation with 10 mM Iodoacetamide for 45 min in the dark at room temperature while shaking at 1,000 rpm. Overnight digestion of the samples was performed by adding 0.5 µg of trypsin to each sample. The following morning, the samples were acidified with neat formic acid (FA) to the final concentration of 1% FA (pH <3).

Digested peptide samples were desalted using in-house packed C18 (3 M) StageTips. C18 StageTips were conditioned sequentially with 100 µl of 100% methanol (MeOH), 100 µl of 50% (v/v) acetonitrile (MeCN) with 0.1% (v/v) FA, and two washes of 100 µl of 0.1% (v/v) FA. Acidified peptides were loaded onto the C18 StageTips and washed twice with 100 µl of 0.1% FA. The peptides were then eluted from the C18 resin using 50 µl of 50% MeCN and 0.1% FA. The desalted peptide samples were snap-frozen and vacuum-centrifuged until completely dry.

### TMT labeling and fractionation of peptides for analysis by LC–MS/MS

Desalted peptides were labeled with TMT16 reagents (Thermo Fisher Scientific). Each peptide sample was resuspended in 80 μl of 50 mM HEPES and labeled with 20 µl of the 25 µg µl^−1^ TMT reagents in MeCN. The samples were then incubated at room temperature for 1 h while shaking at 1,000 rpm. To quench the TMT-labeling reaction, 4 μl of 5% hydroxylamine was added to each sample, followed by a 15-min incubation at room temperature with shaking. TMT-labeled samples were combined and vacuum-centrifuged to dry. The samples were then reconstituted in 200 μl of 0.1% FA and desalted on a C18 StageTip using the previously described protocol. The desalted TMT-labeled combined sample was then dried to completion.

The combined TMT-labeled peptide sample was fractionated by basic reverse-phase fractionation using an in-house packed styrenedivinylbenzene reversed-phase sulfonate (3M) StageTip. A StageTip containing three plugs of styrenedivinylbenzene reversed-phase sulfonate material was prepared and conditioned with 100 μl of 100% MeOH, 100 μl of 50% MeCN and 0.1% FA, and 2× with 100 μl of 0.1% FA. The sample was resuspended in 200 µl of 0.1% FA (pH <3) and loaded onto the conditioned StageTip and eluted in a series of buffers with increasing MeCN concentrations. Six fractions were collected in 20 mM ammonium formate (5%, 10%, 15%, 20%, 25% and 45% MeCN), dried to completion and analyzed by LC–MS/MS.

### Liquid chromatography tandem mass spectrometry

All peptide samples were separated and analyzed on an online LC–MS/MS system, consisting of a Vanquish Neo UPHLC (Thermo Fisher Scientific) coupled to an Orbitrap Exploris 480 (Thermo Fisher Scientific). All peptide fractions were reconstituted in 9 µl of 3% MeCN and 0.1% FA. Four microliters of each fraction was injected onto a microcapillary column (Picofrit with 10 µm tip opening, 75 µm diameter; New Objective, cat. no. PF360-75-10-N-5), packed in-house with 30 cm of C18 silica material (1.5 µm ReproSil-Pur C18-AQ medium; Dr Maisch, cat. no. r119.aq) and heated to 50 °C using column heater sleeves (PhoenixST). Peptides were eluted into the Orbitrap Exploris 480 at a flow rate of 200 nl min^−1^. The basic reverse-phase fractions were run on a 154 min-method. Solvent A comprised 3% acetonitrile and 0.1% FA. Solvent B comprised 90% acetonitrile and 0.1% FA. The LC–MS/MS method used the gradient profile (min, % B): 0:2, 1:6, 122:35, 130:60, 133:90, 143:90, 144:50 and 154:50 (the last two steps were at a 500 nl min^−1^ flow rate).

MS was conducted using a data-dependent acquisition mode, MS1 spectra were measured with a resolution of 60,000, a normalized AGC target of 100%, and a mass range from 350 to 1,800 *m*/*z*. MS2 spectra were acquired for the top 20 most abundant ions per cycle at a resolution of 45,000, an AGC target of 50%, an isolation window of 0.7 *m*/*z* and a normalized collision energy of 32. The dynamic exclusion time was set to 20 s.

### Analysis of mass spectrometry data

MS data were processed using Spectrum Mill (https://proteomics.broadinstitute.org). Spectra in a precursor mass range of 600–6,000 Da with a minimum MS1 signal-to-noise ratio of 25 were retained. In addition, MS1 spectra in a retention time range of ±45 s, or a precursor *m*/*z* tolerance of ±1.4 *m*/*z* were merged. MS/MS searching was performed against a human UniProt database. For searching, fixed modifications were TMT16-Full-Lys modification and carbamidomethylation on cysteine. Variable modifications included acetylation of the protein N terminus, oxidation of methionine and cyclization to pyroglutamic acid. Digestion parameters were set to ‘trypsin allow P’ with an allowance of four missed cleavages. The matching tolerances were set with a minimum matched peak intensity of 30%, precursor and product mass tolerance of ±20 ppm.

Peptide spectrum matches were validated with a maximum false discovery rate (FDR) threshold of 1.2% for precursor charges ranging from +2 to +6. A target protein score of 9 was applied during protein polishing auto-validation to further filter peptide spectrum matches. TMT16 reporter ion intensities were corrected for isotopic impurities using the afRICA correction method in the Spectrum Mill protein or peptide summary module, which utilizes determinant calculations according to Cramer’s rule. Protein quantification and statistical analysis were performed using the Proteomics Toolset for Integrative Data Analysis (Protigy, v.1.0.7; Broad Institute, https://github.com/broadinstitute/protigy). Differential protein expression was evaluated using moderated *t*-tests, with two-sided *P* values calculated to assess significance.

### Quantitative PCR with reverse transcription

Total RNA was extracted from cells using the RNeasy Mini Kit (Qiagen, cat. no. 74106) according to the manufacturer’s instructions. cDNA was synthesized from 1 μg of total RNA using the iScript RT Supermix for RT–qPCR (Bio-Rad Laboratories, cat. no. 1708841) following the manufacturer’s protocol. The synthesized cDNA was diluted 1:10 with nuclease-free water before use. RT–qPCR was performed on a LightCycler 480 II system (Roche Diagnostics) using the LightCycler 480 SYBR Green I Master Mix (Roche Diagnostics, cat. no. 04887352001). Each reaction contained 1 μl of primer mix, 2 μl of diluted cDNA (1:10), 3 μl of nuclease-free water and 5 μl of SYBR Green Master Mix, in a total volume of 10 μl. All reactions were run in triplicate, and relative gene expression levels were calculated using the ΔΔCt method, with target gene expression normalized to housekeeping controls.

### RT–qPCR primers

*PTHLH* Forward 5′-GAACTGGCTCTGCCTGGTTAGA -3′

*PTHLH* Reverse 5′- GTCCTTGGAAGGTCTCTGCTGA-3′

*NDRG1* Forward 5′- CTCCTGCAAGAGTTTGATGTCC -3′

*NDRG1* Reverse 5′- TCATGCCGATGTCATGGTAGG-3′

*IGFBP3* Forward 5′- CGCTACAAAGTTGACTACGAGTC-3′

*IGFBP3* Reverse 5′- GTCTTCCATTTCTCTACGGCAGG-3′

*BNIP3* Forward 5′- TCCTGGGTAGAACTGCACTTC-3′

*BNIP3* Reverse 5′- GCTGGGCATCCAACAGTATTT-3′

*IGFBP4* Forward 5′-ACCCACGAGGACCTCTACATCA -3′

*IGFBP4* Reverse 5′- CACACCAGCACTTGCCACGCT -3′

*IGFBP6* Forward 5′- CACAGGATGTGAACCGCAGAGA-3′

*IGFBP6* Reverse 5′- CACTGAGTCCAGATGTCTACGG-3′

*IGF1* Forward 5′-GCTCTTCAGTTCGTGTGTGGA -3′

*IGF1* Reverse 5′- GCCTCCTTAGATCACAGCTCC-3′

*SFRP4* Forward 5′- ACGAGCTGCCTGTCTATGAC-3′

*SFRP4* Reverse 5′- TGTCTGGTGTGATGTCTATCCAC-3′

*IL6* Forward 5′- AGACAGCCACTCACCTCTTCAG-3′

*IL6* Reverse 5′- TTCTGCCAGTGCCTCTTTGCTG-3′

*CXCL8* Forward 5′- GAGAGTGATTGAGAGTGGACCAC-3′

*CXCL8* Reverse 5′- CACAACCCTCTGCACCCAGTTT-3′

*GDF15* Forward 5′- CAACCAGAGCTGGGAAGATTCG-3′

*GDF15* Reverse 5′- CCCGAGAGATACGCAGGTGCA-3′

*EGLN3* Forward 5′- TCCTGCGGATATTTCCAGAGG-3′

*EGLN3* Reverse 5′- GGTTCCTACGATCTGACCAGAA-3′

*VEGFA* Forward 5′- AGGGCAGAATCATCACGAAGT -3′

*VEGFA* Reverse 5′- AGGGTCTCGATTGGATGGCA -3′

*PTHLH*-V5-ORF Forward 5′- AACGTCGCTGGAGCTCG -3′

*PTHLH*-V5-ORF Reverse 5′- GTGGGTTTGGGATTGGCTTTCC -3′

*UBC* Forward 5′- CTGGAAGATGGTCGTACCCTG -3′

*UBC* Reverse 5′- GGTCTTGCCAGTGAGTGTCT -3′

*ACTB* Forward 5′- CATGTACGTTGCTATCCAGGC-3′

*ACTB* Reverse 5′- CTCCTTAATGTCACGCACGAT -3′

### Colorimetric calcium assay

Serum calcium concentrations were measured using a colorimetric calcium assay kit (Abcam, cat. no. ab102505) according to the manufacturer’s instructions. Briefly, 10 µl of each serum sample was added to the well of a 96-well plate and brought to a total volume of 50 µl with deionized water. Then 90 µl of chromogenic reagent and 60 µl of calcium assay buffer were added to each well, followed by incubation at room temperature in the dark for 5–10 min. Absorbance was measured at 575 nm using a microplate reader. A calcium standard curve was generated with a series of known calcium concentrations, and sample concentrations were calculated based on this curve.

### PTHrP and GDF15 ELISA assay

We measured the PTHrP 1-34 active peptide in cell culture media using the PTHrP (1-34) EIA Kit, extraction-free (Phoenix Pharmaceuticals, cat. no. EK-056-04) and GDF15 using the Human GDF-15 ELISA Kit—Quantikine (R&D Systems, cat. no. DGD150) according to the manufacturer’s instructions. For each assay, 0.3 × 10^6^ cells were seeded per well in a six-well plate. The following day, the media was replaced with 2 ml of DMEM or RPMI containing 1% P–S and 0.5% FBS, and, where noted, the indicated treatment. After 48 h, media were collected and centrifuged at 400*g* for 2 min to pellet any debris, and the supernatant was saved. Simultaneously, cells were lysed in 150 µl of lysis buffer, and total protein concentration was measured. Then 50 µl of conditioned media was used to measure PTHrP (ng ml^−1^) or GDF15 (pg ml^−1^) concentration in units, which was subsequently normalized to the total cellular protein (μg), where total cellular protein = total cellular protein concentration (μg ml^−1^) × 0.15 ml.

### Knock-in FLAG-HA tag at the endogenous HIF2α locus

The generation of 3×FLAG-HA-HIF2α knock-in cells was performed following the protocol published in ref. ^8^, summarized here. SPRI magnetic beads (GE Healthcare, cat. no. 65152105050250) were used for purification of the HDR template synthesized to introduce a 3×FLAG-HA tag at the N terminus of HIF2α. Cas9-ribonucleoprotein complexes were assembled using Alt-R Cas9 Nuclease V3 (stock solution, 62 mM; IDT, cat. no. 1081059), synthetic sgRNA (IDT) and Alt-R Cas9 Electroporation Enhancer (IDT). OSRC-2 cells (2 × 10^5^) were electroporated with Cas9-ribonucleoprotein complexes and HDR template using the 4D-Nucleofector X unit (Lonza) with condition EN138. Post-electroporation, cells were cultured in media supplemented with Alt-R HDR Enhancer V2 (IDT) and refreshed after 16 h. Knock-in efficiency was confirmed by amplicon sequencing or immunoblot analysis 3–4 days later. Single-cell clones were generated for subsequent ChIP–seq assays.

### Chromatin immunoprecipitation

ChIP was performed as described in ref. ^8^. Briefly, 1 × 10^7^ OSRC-2 cells were crosslinked using 1% formaldehyde, which was then quenched with glycine. Crosslinked cells were lysed in SDS lysis buffer (1% SDS, 10 mM EDTA, 50 mM Tris–HCl, pH 8.0) supplemented with protease inhibitors and 5 mM sodium butyrate. Chromatin was fragmented by sonication (Covaris E220) to an average size of ~200–500 bp. For ChIP–seq, 40 µg of chromatin was immunoprecipitated using 5 µg FLAG antibody (Sigma-Aldrich, cat. no. F1804). Antibody-bead conjugates were prepared using protein G magnetic beads (Life Technologies, cat. no. 10004D) and washed extensively. Bead-bound chromatin was washed sequentially with RIPA 0, RIPA 0.3 and LiCl buffers before elution in SDS elution buffer. DNA was reverse crosslinked at 65 °C and purified using the MinElute PCR Purification Kit (Qiagen, cat. no. 28004). ChIP–seq libraries were prepared using the Swift DNA Library Prep Kit and sequenced on an Illumina NextSeq 500 platform. Peak calling and differential binding analyses were performed using MACS2 and DEseq2 in the CoBRA pipeline as described in ref. ^8^.

### RNA-seq sample library preparation and sequencing

For 72 h PT2399 treatment and sg*EPAS1* experiments, RNA-seq was done as described in ref. ^8^. In brief, ribosomal RNA-depleted libraries were prepared from 100 ng of total RNA using the KAPA RNA HyperPrep Kit with RiboErase (Roche, cat. no. 08098131702). RNA was fragmented at 94 °C for 8 min, followed by first- and second-strand cDNA synthesis. cDNA fragments were end-repaired, adenylated at the 3′ ends, and ligated to universal adapters. Indexed libraries were enriched by 14 cycles of PCR using a Beckman Coulter Biomek i7 Automated Workstation. Libraries were quantified using a Qubit 4 Fluorometer and Agilent TapeStation 4200, pooled in an equimolar ratio, and shallowly sequenced on an Illumina MiSeq to assess quality. Final sequencing was conducted with paired-end 150-bp reads on an Illumina NovaSeq 6000 at the Dana-Farber Cancer Institute Molecular Biology Core Facilities.

For 24 h and 48 h PT2399 treatment experiments, cells were seeded at a density of 1 × 10^6^ cells per 100 mm tissue culture dish (Corning, cat. no. 353003) and, in triplicate, treated with either DMSO or 2 µM PT2399 for 24 h or 48 h. Following treatment, the cells were rinsed twice with ice-cold DPBS (Gibco, cat. no. 14190094) and scraped into 1 ml of ice-cold DPBS. The cell suspension was centrifuged at 1,200*g* for 5 min at 4 °C to pellet the cells. The pellets were then flash-frozen in liquid nitrogen and stored at −80 °C until processing.

For RNA extraction, the Qiagen RNeasy Mini Kit (cat. no. 74106) was used. RNA quantity and quality were measured on a NanoDrop spectrophotometer (Thermo Fisher Scientific, cat. no. ND-8000-GL). The samples were subsequently sent to GENEWIZ for library preparation and sequencing. Before library construction, normalization was achieved by adding the ERCC RNA Spike-In Mix kit (Thermo Fisher Scientific, cat. no. 4456740) according to the manufacturer’s protocol.

Library construction began with the enrichment of mRNA using oligo(dT) beads. The enriched mRNA was then fragmented by incubation with NEBNext First Strand Synthesis Reaction Buffer at 94 °C for 15 min. First- and second-strand cDNA syntheses were performed next, followed by end repair and 3’ adenylation of the cDNA fragments. Universal adapters were ligated, and indexed PCR amplification (with a limited number of cycles) was carried out to enrich the library. Library quality was verified using an Agilent TapeStation (Agilent Technologies, cat. no. G2991BA) and quantified with both a Qubit 2.0 Fluorometer and quantitative PCR (KAPA Biosystems, cat. no. KK4824).

Clusters were generated on lanes of a single flow cell, which was then loaded onto an Illumina HiSeq instrument (4000 or equivalent) per the manufacturer’s instructions. Sequencing was performed using a 2 × 150 bp paired-end configuration. Image analysis and base calling were executed with HiSeq Control Software, and the raw.bcl files were converted to FASTQ files and demultiplexed using Illumina’s bcl2fastq 2.17 software, allowing for one mismatch in index sequence identification.

The paired-end FASTQ files were aligned to the hg38 genome using HISAT2. Gene-level read counts were obtained with featureCounts and normalized using DESeq2, which modeled the average gene expression in each sample with a negative binomial distribution. Differential expression analysis between PT2399-treated (24 h or 48 h) and DMSO-treated samples was performed using a Wald test provided by DESeq2, and corresponding plots were generated to illustrate these differences.

### PRO-seq sample preparation and analysis

Pro-seq was done as described in ref. ^8^. Briefly, cells were harvested, permeabilized and flash-frozen in freezing buffer. Nascent RNA was labeled using biotin-11-nucleoside triphosphates in nuclear run-on assays and purified using streptavidin beads. Following fragmentation and adapter ligation, reverse transcription was performed to generate cDNA, which was amplified to construct sequencing libraries. Libraries were sequenced using the Illumina NovaSeq platform, and the data were analyzed with scripts developed by the AdelmanLab. Transcription start sites were annotated using Ensembl GTF files, and browser snapshots were generated with IGV for visualization.

### Polysome-seq sample preparation

Polysome-seq was done as described in ref. ^8^. Briefly, 1 × 10^7^ to 2 × 10^7^ OSRC-2 cells were washed with ice-cold PBS and lysed on ice using polysome extraction buffer (25 mM HEPES pH 7.5, 5 mM MgCl_2_, 100 mM KCl, 2 mM DTT, 1% Triton X-100, 0.1 mg ml^−1^ cycloheximide, 0.05 U μl^−1^ RNase inhibitor and 1× EDTA-free protease inhibitor cocktail). Lysates were homogenized with a glass Dounce homogenizer and cleared by centrifugation (10,000*g*, 5 min, 4 °C). The supernatant was loaded onto 10–50% sucrose gradients and centrifuged (236,000*g*, 3 h, 4 °C, SW41Ti rotor). Gradients were fractionated, and polysome-containing fractions (all heavier than the monosome peak) were pooled for RNA purification using TRIzol LS, followed by sequential isopropanol and ethanol precipitations. RNA quality was verified (RNA integrity number >9) using the Agilent RNA TapeStation, and sequencing libraries were prepared using the SMARTer Stranded Total RNA Sample Prep Kit—HI Mammalian. Final libraries were quantified with Qubit HS DNA assays and an Agilent TapeStation, pooled with unique dual indexes, and sequenced on an Illumina NovaSeq 6000.

### Differential gene expression analysis for RNA-seq and polysome-seq

Sequenced reads were aligned to the UCSC hg38 reference genome assembly and gene counts were quantified using STAR (v.2.7.3a)^65^ and Salmon^66^. Differential gene expression testing was performed by DESeq2 (v.1.22.1)^67^. RNA-seq analysis was performed using the VIPER snakemake pipeline^68^.

### Mouse xenograft models

All experimental procedures involving orthotopic and subcutaneous xenografts were approved by the Dana-Farber Cancer Institute (protocol no. 04-019) or the University of Massachusetts Chan Medical School Institutional (protocol no. 202200072) Animal Care and Use Committees. Mice were housed in a pathogen-free facility under a 12-h light and 12-h dark cycle, with ambient temperature maintained at 23–25 °C and relative humidity at 40–60%, with food and water provided ad libitum. In accordance with institutional animal care guidelines, the maximum permitted tumor size was 2 cm in the largest diameter or 10% of body weight, and this limit was not exceeded in any experiment. All in vivo experiments were performed using female mice aged 8–10 weeks. Female mice were selected based on previous experience with the OSRC-2 xenograft model, which showed consistent tumor engraftment, and because they are generally easier to handle, reducing stress-related variability. Female NCr nude mice (NCRNU-F sp/sp CrTac:NCr-*Foxn*1^nu^) were obtained from Taconic and female NOD Cg-*Prkdc*^scid^ *Il2rg*t^m1Wjl^/SzJ (NGS) mice were obtained from The Jackson Laboratory. These strains are immunodeficient and were maintained on their standard genetic backgrounds. To establish orthotopic xenografts, 1 × 10^6^ 786-O-Fluc cells or 0.5 × 10^6^ OSRC-2-Fluc cells were injected into the parenchyma of the left kidney of nude mice, as described by us previously^26^. For subcutaneous xenografts, 5 × 10^6^ OSRC-2-Fluc cells were injected into the right flank of nude mice or 6 × 10^6^ RXF393-Fluc cells were injected subcutaneously into both the right and left flanks of nude mice or NSG mice. For the data in Fig. 1 and Extended Data Fig. 6, tumors were allowed to form, and mice body weights were monitored. When a mouse lost >10% of its body weight, blood was drawn to collect serum for calcium measurement. Mice were then randomized to receive either 45 mg kg^−1^ PT2399 (formulated in 10% ethanol, 30% PEG 400 and 60% water containing 0.5% methylcellulose and 0.5% Tween 80) or vehicle alone, administered daily by oral gavage for 6 days. Body weight was monitored daily. For the ZOL experiments in Extended Data Fig. 4h–n, OSRC-2 tumor-bearing mice were randomized into three treatment arms: vehicle, PT2399 (45 mg kg^−1^ daily oral gavage), or ZOL (120 μg kg^−1^ intraperitoneally every other day). For the data in Extended Data Fig. 1a–c, PT2399 began 4 weeks after tumor cell implantation and was dosed at 30 mg kg^−1^ daily by oral gavage. In the experiments with DOX-inducible, mice were fed a normal chow diet (−DOX) or, where indicated, a DOX 2,000 ppm green diet (TestDiet, cat. no 1811824). At the study endpoint, mice were euthanized using CO_2_. Tumors were then harvested and weighed. A portion of each tumor was frozen at −80 °C, while the remainder was fixed in 10% paraformaldehyde for histological analysis.

For the radiological and histological characterization of the cachexia in OSRC-2 cells model, NCr nude female mice were purchased from Taconic (NCRNU-F sp/sp CrTac:NCr-*Foxn*1^nu^) and were allowed to acclimatize for a week with ad libitum access to food and water. Mice were implanted with 5 × 10^6^ OSRC-2 cells (or sham controls for NTB) subcutaneously and weighed every other day. Tumor-bearing mice were randomized into vehicle or PT2399 treatment (30 mg kg^−1^) arms and received one dose daily by oral gavage for a total of 17 days. Body scans were performed on an Echo Medical Systems 1H-MRS by the UMass Metabolic Disease Research Center to measure the fat and lean masses of each animal right before the first dose of drug and 17 days into treatment. Mice were housed in TSE metabolic cages (TSE Systems) in the Metabolic Disease Research Center facility, where daily food intake was monitored over a 3-day period. After the last treatment, mice were euthanized and tumor, iWAT, eWAT, brown adipose tissue, gastrocnemius and quadriceps tissues were collected, weighed and processed for downstream analysis. For hematoxylin and eosin and immunofluorescent staining, dissected tissue was immediately fixed in zinc formalin, processed, embedded in paraffin, sectioned and mounted. Immunofluorescent sections were deparaffinized and rehydrated, followed by antigen retrieval and overnight incubation with Ucp1 (Sigma, cat. no. U6382) primary antibody at 4 °C at 1:1,000. Images were acquired using an EVOS M5000 fluorescent microscope and quantification of percent area was performed using ImageJ software.

### *PTHLH* mRNA expression and copy number analysis

The gene expression files for each TCGA sample (*.rna_seq.augmented_star_gene_counts.tsv) were downloaded from the GDC Data Portal, and TPM values were extracted. Only data from primary tumor samples were included in the analysis. Copy number variation data for PTHLH were retrieved from the ‘PanCancer Atlas’ dataset in cBioPortal. Copy numbers corresponding to the PTHLH gene were classified as follows: copy number (cn) = 2 (amplification), cn = 1 (gain), cn = 0 (diploid), cn = −1 (heterozygous deletion) and cn = −2 (homozygous deletion). Data were restricted to primary tumor samples. To analyze the relationship between copy number and gene expression levels for genes on chromosome 12p in kidney renal clear cell carcinoma, samples were grouped based on copy number variation status (amplification or gain versus no copy number change), and the mean log_2_(fold change) in gene expression was calculated. Statistical differences between groups were assessed using the unpaired Wilcoxon rank sum test. In addition, relative expression levels normalized to *ACTB* were calculated for samples with amplification or gain, and the mean values were reported.

### Clinical data for belzutifan, ICI and VEGF TKI

Plasma samples were obtained for patients with advanced ccRCC treated at the Dana-Farber Cancer Institute under protocol no. 01-130, which was approved by the Dana-Farber/Harvard Cancer Center Institutional Review Board. Matched pre- and post-treatment samples (250 µl each) were collected from patients receiving either the HIF2 inhibitor (belzutifan), ICIs or VEGF TKIs. Plasma PTHrP levels were measured using an immunochemiluminometric assay at the Mayo Clinic (test id: PTHRP). Clinical data, including baseline characteristics, body weight and albumin-corrected calcium levels at the time therapy was initiated, 1 month and 3 months thereafter were retrospectively collected. Variations in PTHrP levels, corrected calcium and body mass index were evaluated using paired Wilcoxon signed-rank tests. Both male and female patients with RCC were included in this study and were reported in Extended Data Table 1. Sex was determined from medical records as sex assigned at birth. Analyses were performed disaggregated by sex where relevant and included in the source data.

### Clinical data for NKT2152

Plasma samples (in K2 EDTA tubes) were collected from 2021 to 2024 in the dose-escalation part of a phase I study of NKT2152, an HIF2 inhibitor, in patients with advanced or metastatic ccRCC (NCT05119335). To be eligible for this study patients had to be aged 18 years or older and with locally advanced or metastatic ccRCC and to have exhausted available standard therapy as determined by the investigator. Sixty subjects were enrolled in the dose-escalation part, among whom 45 had plasma samples for the PTHrP assay and data analysis. PTHrP level was measured at Mayo Clinic with a immunochemiluminometric assay (test id: PTHRP). Sample shipping was at −20 °C with dry ice; sample storage was at −70 °C. There was a freeze–thaw cycle for the transfer of the plasma samples stored in a 2.0-ml Cryovial tube to a 5.0-ml test tube before the test.

For PTHrP results <0.4 pmol l^−1^, 0.2 ($\frac{1}{2}$ of limit of quantitation) was used for plots. Windowing algorithm and Last Observation Carried Forward were applied for plotting of longitudinal body weight and calcium level changes. The clinical data cutoff date was 16 June 2024. Both male and female patients with RCC were included in this study. Sex was determined from medical records as sex assigned at birth and is reported in Extended Data Table 1. Analyses were performed disaggregated by sex where relevant and included in the source data.

### Statistical analysis

Statistical analyses were performed using GraphPad Prism (v.9) and R (v.4.3.0, RStudio 2023.9.1.494, with packages tidyverse v.2.0.0, ggpubr v.0.6.0, ggsci v.3.0.0, and table1 v.1.4.3). Data are presented as mean ± s.e.m. Normality was visually assessed, and appropriate statistical tests were selected accordingly. Data distribution was assumed to be normal, but this was not formally tested, and equal variances were not formally tested. Data distribution is shown in the figures as individual data points wherever possible.

For comparisons between two groups, unpaired two-tailed Student’s *t*-test or unpaired Wilcoxon rank sum test was used, depending on data distribution. For paired data comparisons, the paired Wilcoxon signed-rank test was applied. For multiple group comparisons, two-way analysis of variance was performed. Kaplan–Meier survival analyses were assessed using the log-rank test.

Scatterplots were generated to visualize the relationship between specific tissue mass measurements and pretreatment weights. ANCOVA was utilized to address the relationship of specific mass measurements and treatment group to specifically compare vehicle to PT2399, when the similar slope assumption was met. All models were adjusted for pretreatment body weight. A heteroskedasticity-consistent variance-covariance matrix was used to calculate robust estimates.

For omics data analysis, RNA-seq differential expression analysis was performed using DESeq2, with statistical significance determined by the two-sided Wald test. ChIP–seq peak calling was conducted using MACS2, and proteomics data were normalized using Spectrum Mill. Analyses of clinical dataset of NKT2152 were conducted using SAS (v.9.4).

All statistical details, including the number of samples (*n*), statistical tests used and significance thresholds are provided in the figure legends and in the relevant Methods sections. *n* represents biological replicates, not technical replicates. For in vivo studies, *n* corresponds to individual animals; for in vitro studies, *n* corresponds to independent biological experiments.

Mice were randomly assigned to treatment groups, and blinding was applied for histological assessments. For all other analyses, data collection and analysis were not performed blind to the conditions of the experiments. For in vitro studies, treatments were randomly allocated across plates processed in parallel. All experiments were replicated at least twice. Statistical significance was set at two-sided *P* ≤ 0.05, with exact *P* values reported except calculation of the empiric *P* value in Extended Data Fig. 7g–m was one-sided. No data were excluded from analyses.

No statistical methods were used to predetermine sample sizes, but the sample sizes used in this study are similar to those reported in previous publications investigating xenograft models and molecular analyses in kidney cancer and cachexia^18,26^.

### Reporting summary

Further information on research design is available in the Nature Portfolio Reporting Summary linked to this article.

## Online content

Any methods, additional references, Nature Portfolio reporting summaries, source data, extended data, supplementary information, acknowledgements, peer review information; details of author contributions and competing interests; and statements of data and code availability are available at 10.1038/s41591-025-04054-2.

## Supplementary information

Reporting Summary

## Source data

Source Data Figs. 1 and 2 and Extended Data Figs. 1 and 3–6 Unprocessed western blots.

## Figures and Tables

**Extended Data Table 1:**
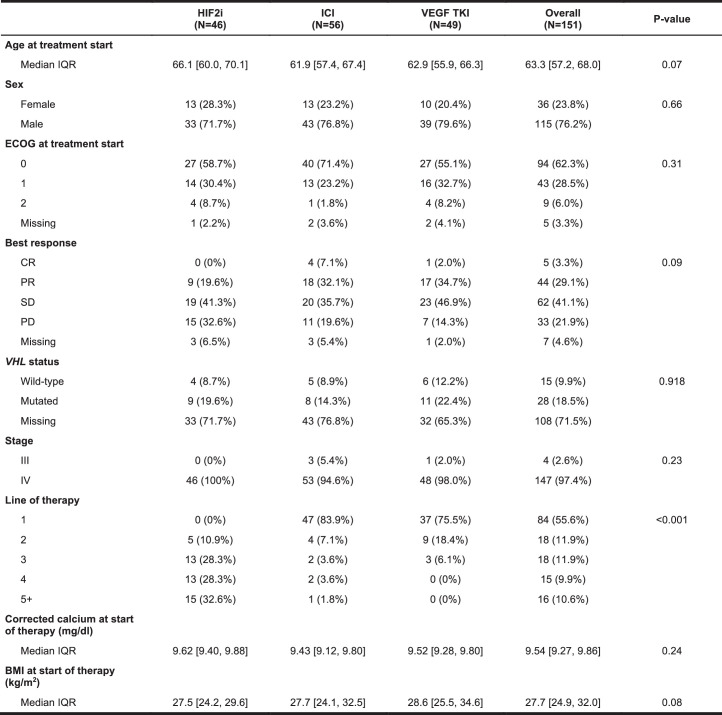
Baseline characteristic of the patients with clear-cell renal cell carcinoma from the Dana-Farber cohort

CR: complete response, ECOG: Eastern Cooperative Oncology Group, HIF2i: HIF-2α inhibitors, ICI: immune checkpoint inhibitors, IQR: interquartile range, PD: progressive disease, PR: partial response, SD: stable disease, VEGF TKI: vascular endothelial growth factor tyrosine kinase inhibitor, *VHL*: Von-Hippel Lindau gene

Statistical comparisons between the 3 treatment groups were performed using the two-sided Chi-square test for categorical variables and the two-sided Kruskal-Wallis test for continuous variables.

**Extended Data Table 2:**
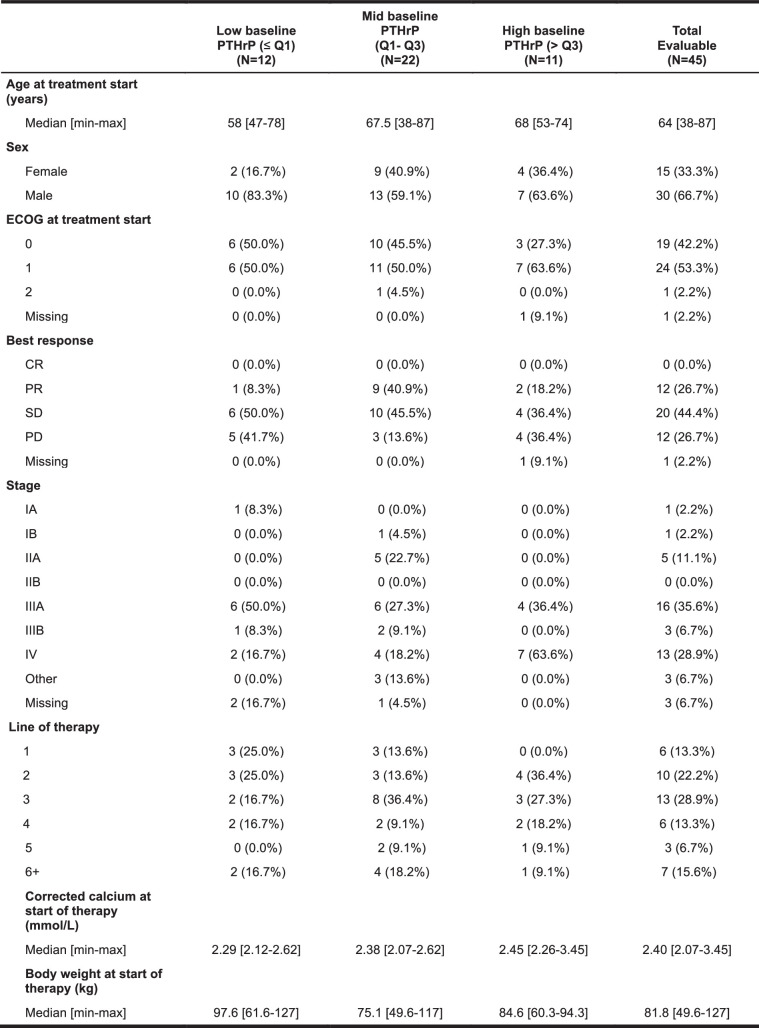
Baseline characteristic of the patients with clear-cell renal cell carcinoma from the Nikang cohort

CR: complete response, ECOG: Eastern Cooperative Oncology Group, PD: progressive disease, PR: partial response, PTHrP: parathyroid hormone-related protein, Q1: first quartile, Q2: second quartile, Q3: third quartile, SD: stable disease.

## Data Availability

There are no restrictions on data availability for this study. The original mass spectra and protein sequence databases used for searches have been deposited in the public proteomics repository MassIVE under accession MSV000097181 (http://massive.ucsd.edu). The processed datasets, data tables from Figs. 2 and 3, Extended Data Fig. 3, the uncropped western blot scans and the sex-stratified analyses of belzutifan and NKT2152 clinical data in RCC patients for Fig. 6 and Extended Data Fig. 8 are available via Zenodo at 10.5281/zenodo.14902967 (ref. ^69^). All raw and processed sequencing datasets have been deposited in GEO. RNA-seq (24 h and 48 h PT2399 treatment), ChIP–seq and PRO-seq data are available under accession GSE277046. RNA-seq (72 h PT2399 treatment and sgEPAS1) data are available under accession GSE289579. Polysome-seq data are available under accession GSE289581. Source data are provided with this paper.

## Code availability

The code used to analyze ChIP–seq, RNA-seq (24 h and 48 h PT2399 treatment) and PRO-seq datasets has been deposited in the Kaelin Lab GitHub repository and is publicly available at https://github.com/kaelinlabdfci/HIF2a_CCND1_ccRCC. The code used to analyze TCGA datasets has been deposited in the Kaelin Lab GitHub repository and is publicly available at https://github.com/kaelinlabdfci/HIF2-RCC-Cachexia.

## References

[1] Cancer stat facts: kidney and renal pelvis cancer. *National Cancer Institute Surveillance, Epidemiology and End Results Program* https://seer.cancer.gov/statfacts/html/kidrp.html (2025).

[2] Sung, H. et al. Global cancer statistics 2020: GLOBOCAN estimates of incidence and mortality worldwide for 36 cancers in 185 countries. *CA Cancer J. Clin.* **71**, 209–249 (2021). 33538338 10.3322/caac.21660

[3] Moch, H., Cubilla, A. L., Humphrey, P. A., Reuter, V. E. & Ulbright, T. M. The 2016 WHO classification of tumours of the urinary system and male genital organs–part A: renal, penile, and testicular tumours. *Eur. Urol.* **70**, 93–105 (2016). 26935559 10.1016/j.eururo.2016.02.029

[4] Latif, F. et al. Identification of the von Hippel–Lindau disease tumor suppressor gene. *Science* **260**, 1317–1320 (1993). 8493574 10.1126/science.8493574

[5] Kaelin, W. G. Jr. The von Hippel–Lindau tumour suppressor protein: O_2_ sensing and cancer. *Nat. Rev. Cancer* **8**, 865–873 (2008). 18923434 10.1038/nrc2502

[6] Gordan, J. D. et al. HIF-alpha effects on c-Myc distinguish two subtypes of sporadic VHL-deficient clear cell renal carcinoma. *Cancer Cell* **14**, 435–446 (2008). 19061835 10.1016/j.ccr.2008.10.016 PMC2621440

[7] Motzer, R. J. et al. Sunitinib versus interferon alfa in metastatic renal-cell carcinoma. *N. Engl. J. Med.* **356**, 115–124 (2007). 17215529 10.1056/NEJMoa065044

[8] Jiang, Q. et al. HIF regulates multiple translated endogenous retroviruses: implications for cancer immunotherapy. *Cell* **188**, 1807–1827 (2025). 40023154 10.1016/j.cell.2025.01.046 PMC11988688

[9] Courtney, K. D. et al. HIF-2 complex dissociation, target inhibition, and acquired resistance with PT2385, a first-in-class HIF-2 inhibitor, in patients with clear cell renal cell carcinoma. *Clin. Cancer Res.* **26**, 793–803 (2020). 31727677 10.1158/1078-0432.CCR-19-1459 PMC7024660

[10] Jonasch, E. et al. Belzutifan for renal cell carcinoma in von Hippel–Lindau Disease. *N. Engl. J. Med.* **385**, 2036–2046 (2021). 34818478 10.1056/NEJMoa2103425 PMC9275515

[11] Fallah, J. et al. FDA approval summary: belzutifan for patients with advanced renal cell carcinoma. *Clin. Cancer Res.* **30**, 5003–5008 (2024). 39248780 10.1158/1078-0432.CCR-24-1199 PMC11567780

[12] Palapattu, G. S., Kristo, B. & Rajfer, J. Paraneoplastic syndromes in urologic malignancy: the many faces of renal cell carcinoma. *Rev. Urol.* **4**, 163–170 (2002). 16985675 PMC1475999

[13] Kim, H. L. et al. Paraneoplastic signs and symptoms of renal cell carcinoma: implications for prognosis. *J. Urol.* **170**, 1742–1746 (2003). 14532767 10.1097/01.ju.0000092764.81308.6a

[14] Sun, R. et al. Prognostic impact of paraneoplastic syndromes on patients with non-metastatic renal cell carcinoma undergoing surgery: results from Canadian Kidney Cancer information system. *Can. Urol. Assoc. J.* **15**, 132–137 (2021). 33007184 10.5489/cuaj.6833 PMC8021432

[15] Ghosh, M. C. et al. Therapeutic inhibition of HIF-2alpha reverses polycythemia and pulmonary hypertension in murine models of human diseases. *Blood* **137**, 2509–2519 (2021). 33512384 10.1182/blood.2020009138 PMC8109019

[16] Baracos, V. E., Martin, L., Korc, M., Guttridge, D. C. & Fearon, K. C. H. Cancer-associated cachexia. *Nat. Rev. Dis. Prim.* **4**, 17105 (2018). 29345251 10.1038/nrdp.2017.105

[17] von Haehling, S., Anker, M. S. & Anker, S. D. Prevalence and clinical impact of cachexia in chronic illness in Europe, USA, and Japan: facts and numbers update 2016. *J. Cachexia Sarcopenia Muscle* **7**, 507–509 (2016). 27891294 10.1002/jcsm.12167 PMC5114624

[18] Kir, S. et al. Tumour-derived PTH-related protein triggers adipose tissue browning and cancer cachexia. *Nature* **513**, 100–104 (2014). 25043053 10.1038/nature13528 PMC4224962

[19] Kir, S. et al. PTH/PTHrP receptor mediates cachexia in models of kidney failure and cancer. *Cell Metab.* **23**, 315–323 (2016). 26669699 10.1016/j.cmet.2015.11.003 PMC4749423

[20] Deans, C. et al. Serum parathyroid hormone-related peptide is associated with systemic inflammation and adverse prognosis in gastroesophageal carcinoma. *Cancer* **103**, 1810–1818 (2005). 15800880 10.1002/cncr.20972

[21] Iguchi, H., Aramaki, Y., Maruta, S. & Takiguchi, S. Effects of anti-parathyroid hormone-related protein monoclonal antibody and osteoprotegerin on PTHrP-producing tumor-induced cachexia in nude mice. *J. Bone Miner. Metab.* **24**, 16–19 (2006). 16369893 10.1007/s00774-005-0640-5

[22] Hong, N. et al. Serum PTHrP predicts weight loss in cancer patients independent of hypercalcemia, inflammation, and tumor burden. *J. Clin. Endocrinol. Metab.* **101**, 1207–1214 (2016). 26765580 10.1210/jc.2015-3785

[23] Iresjo, B. M., Kir, S. & Lundholm, K. Parathyroid hormone related protein (PTHrP) in patients with pancreatic carcinoma and overt signs of disease progression and host tissue wasting. *Transl. Oncol.* **36**, 101752 (2023). 37540958 10.1016/j.tranon.2023.101752 PMC10407952

[24] Chen, W. et al. Targeting renal cell carcinoma with a HIF-2 antagonist. *Nature* **539**, 112–117 (2016). 27595394 10.1038/nature19796 PMC5340502

[25] Cho, H. et al. On-target efficacy of a HIF-2alpha antagonist in preclinical kidney cancer models. *Nature* **539**, 107–111 (2016). 27595393 10.1038/nature19795 PMC5499381

[26] Stransky, L. A. et al. Sensitivity of VHL mutant kidney cancers to HIF2 inhibitors does not require an intact p53 pathway. *Proc. Natl Acad. Sci. USA* **119**, e2120403119 (2022). 35357972 10.1073/pnas.2120403119 PMC9168943

[27] Shirole, N. H. et al. Requirement for cyclin D1 underlies cell-autonomous HIF2 dependence in kidney cancer. *Cancer Discov.* **15**, 1484–1504 (2025). 40178040 10.1158/2159-8290.CD-24-1378 PMC12223508

[28] Ricquier, D. Uncoupling protein 1 of brown adipocytes, the only uncoupler: a historical perspective. *Front. Endocrinol.* **2**, 85 (2011). 10.3389/fendo.2011.00085 PMC3355862 22649389

[29] Droujinine, I. A. et al. Proteomics of protein trafficking by in vivo tissue-specific labeling. *Nat. Commun.* **12**, 2382 (2021). 33888706 10.1038/s41467-021-22599-x PMC8062696

[30] Suriben, R. et al. Antibody-mediated inhibition of GDF15-GFRAL activity reverses cancer cachexia in mice. *Nat. Med.* **26**, 1264–1270 (2020). 32661391 10.1038/s41591-020-0945-x

[31] Agca, S. & Kir, S. The role of interleukin-6 family cytokines in cancer cachexia. *FEBS J.* **291**, 4009–4023 (2024). 38975832 10.1111/febs.17224

[32] Manisterski, M., Golan, M., Amir, S., Weisman, Y. & Mabjeesh, N. J. Hypoxia induces PTHrP gene transcription in human cancer cells through the HIF-2alpha. *Cell Cycle* **9**, 3723–3729 (2010). 20890122

[33] Messai, Y. et al. ITPR1 protects renal cancer cells against natural killer cells by inducing autophagy. *Cancer Res.* **74**, 6820–6832 (2014). 25297632 10.1158/0008-5472.CAN-14-0303

[34] Rikimaru, K., Matsumoto, F., Hayashi, E., Bando, H. & Nagayama, M. Evaluation of serum concentration of parathyroid hormone-related protein and its implication in hypercalcemia in squamous cell carcinoma of the head and neck. *Int. J. Oral Maxillofac. Surg.* **24**, 365–368 (1995). 8627105 10.1016/s0901-5027(05)80494-1

[35] Suva, L. J. et al. A parathyroid hormone-related protein implicated in malignant hypercalcemia: cloning and expression. *Science* **237**, 893–896 (1987). 3616618 10.1126/science.3616618

[36] Morony, S. et al. The inhibition of RANKL causes greater suppression of bone resorption and hypercalcemia compared with bisphosphonates in two models of humoral hypercalcemia of malignancy. *Endocrinology* **146**, 3235–3243 (2005). 15845617 10.1210/en.2004-1583

[37] Beroukhim, R. et al. Patterns of gene expression and copy-number alterations in von-Hippel Lindau disease-associated and sporadic clear cell carcinoma of the kidney. *Cancer Res.* **69**, 4674–4681 (2009). 19470766 10.1158/0008-5472.CAN-09-0146 PMC2745239

[38] Pitarresi, J. R. et al. PTHrP drives pancreatic cancer growth and metastasis and reveals a new therapeutic vulnerability. *Cancer Discov.* **11**, 1774–1791 (2021). 33589425 10.1158/2159-8290.CD-20-1098 PMC8292165

[39] Broad DepMap. DepMap 24Q4 public. *figshare*10.25452/figshare.plus.27993248.v1 (2024).

[40] Ashrafzadeh-Kian, S., Bornhorst, J. & Algeciras-Schimnich, A. Development of a PTHrP chemiluminescent immunoassay to assess humoral hypercalcemia of malignancy. *Clin. Biochem.* **105–106**, 75–80 (2022). 35429486 10.1016/j.clinbiochem.2022.04.005

[41] Lu, J. et al. NKT2152: a highly potent HIF2α inhibitor and its therapeutic potential in solid tumors beyond ccRCC. *Cancer Res.* **82**, 6330 (2022).

[42] Yao, M. et al. Tumor signatures of PTHLH overexpression, high serum calcium, and poor prognosis were observed exclusively in clear cell but not non clear cell renal carcinomas. *Cancer Med.* **3**, 845–854 (2014). 24861371 10.1002/cam4.270 PMC4303152

[43] Danilin, S. et al. von Hippel–Lindau tumor suppressor gene-dependent mRNA stabilization of the survival factor parathyroid hormone-related protein in human renal cell carcinoma by the RNA-binding protein HuR. *Carcinogenesis* **30**, 387–396 (2009). 19056930 10.1093/carcin/bgn275

[44] Ueno, M., Tokonabe, S., Kuroda, I., Tsukamoto, T. & Deguchi, N. Hypercalcemia upon recurrence of renal cell carcinoma producing parathyroid hormone-related protein. *Scand. J. Urol. Nephrol.* **37**, 265–268 (2003). 12775288 10.1080/00365590310008172

[45] Sourbier, C. & Massfelder, T. Parathyroid hormone-related protein in human renal cell carcinoma. *Cancer Lett.* **240**, 170–182 (2006). 16223565 10.1016/j.canlet.2005.08.020

[46] Baumgarten, A. J., Fiebig, H. H. & Burger, A. M. Molecular analysis of xenograft models of human cancer cachexia–possibilities for therapeutic intervention. *Cancer Genomics Proteomics* **4**, 223–231 (2007). 17878525

[47] Wei, G. et al. The thermogenic activity of adjacent adipocytes fuels the progression of ccRCC and compromises anti-tumor therapeutic efficacy. *Cell Metab.* **33**, 2021–2039 (2021). 34508696 10.1016/j.cmet.2021.08.012

[48] Bhalerao, N. et al. Pancreatic cancer cachexia is mediated by PTHrP-driven disruption of adipose de novo lipogenesis. Preprint at *bioRxiv*10.1101/2025.06.03.657464 (2025).

[49] Liu, X. et al. Activation of GPR81 by lactate drives tumour-induced cachexia. *Nat. Metab.* **6**, 708–723 (2024). 38499763 10.1038/s42255-024-01011-0 PMC11052724

[50] Rohm, M. et al. An AMP-activated protein kinase-stabilizing peptide ameliorates adipose tissue wasting in cancer cachexia in mice. *Nat. Med.* **22**, 1120–1130 (2016). 27571348 10.1038/nm.4171

[51] Li, J. et al. PTHrP drives breast tumor initiation, progression, and metastasis in mice and is a potential therapy target. *J. Clin. Invest.* **121**, 4655–4669 (2011). 22056386 10.1172/JCI46134 PMC3225988

[52] Sato, K. et al. Passive immunization with anti-parathyroid hormone-related protein monoclonal antibody markedly prolongs survival time of hypercalcemic nude mice bearing transplanted human PTHrP-producing tumors. *J. Bone Miner. Res.* **8**, 849–860 (1993). 8352067 10.1002/jbmr.5650080711

[53] Burton, P. B., Moniz, C. & Knight, D. E. Parathyroid hormone related peptide can function as an autocrine growth factor in human renal cell carcinoma. *Biochem. Biophys. Res. Commun.* **167**, 1134–1138 (1990). 2322262 10.1016/0006-291x(90)90641-y

[54] Esbrit, P. & Egido, J. The emerging role of parathyroid hormone-related protein as a renal regulating factor. *Nephrol. Dial. Transpl.* **15**, 1109–1111 (2000). 10.1093/ndt/15.8.1109 10910428

[55] Massfelder, T. et al. Parathyroid hormone-related protein is an essential growth factor for human clear cell renal carcinoma and a target for the von Hippel–Lindau tumor suppressor gene. *Cancer Res.* **64**, 180–188 (2004). 14729622 10.1158/0008-5472.can-03-1968

[56] Talon, I. et al. Antitumor effect of parathyroid hormone-related protein neutralizing antibody in human renal cell carcinoma in vitro and in vivo. *Carcinogenesis* **27**, 73–83 (2006). 16081513 10.1093/carcin/bgi203

[57] Feldser, D. et al. Reciprocal positive regulation of hypoxia-inducible factor 1alpha and insulin-like growth factor 2. *Cancer Res.* **59**, 3915–3918 (1999). 10463582

[58] Lee, J. Y. et al. Effects of serum albumin, calcium levels, cancer stage and performance status on weight loss in parathyroid hormone-related peptide positive or negative patients with cancer. *Endocrinol. Metab.* **33**, 97–104 (2018). 10.3803/EnM.2018.33.1.97 PMC5874202 29589391

[59] Fujimoto-Ouchi, K., Onuma, E., Shirane, M., Mori, K. & Tanaka, Y. Capecitabine improves cancer cachexia and normalizes IL-6 and PTHrP levels in mouse cancer cachexia models. *Cancer Chemother. Pharmacol.* **59**, 807–815 (2007). 17009035 10.1007/s00280-006-0338-y

[60] Scheuermann, T. H. et al. Isoform-selective and stereoselective inhibition of hypoxia inducible factor-2. *J. Med. Chem.* **58**, 5930–5941 (2015). 26226049 10.1021/acs.jmedchem.5b00529

[61] Bui, B. P., Nguyen, P. L., Lee, K. & Cho, J. Hypoxia-inducible factor-1: a novel therapeutic target for the management of cancer, drug resistance, and cancer-related pain. *Cancers* **14**, 6054 (2022). 36551540 10.3390/cancers14246054 PMC9775408

[62] Groarke, J. D. et al. Ponsegromab for the treatment of cancer cachexia. *N. Engl. J. Med.* **391**, 2291–2303 (2024). 39282907 10.1056/NEJMoa2409515

[63] Ran, F. A. et al. Genome engineering using the CRISPR–Cas9 system. *Nat. Protoc.* **8**, 2281–2308 (2013). 24157548 10.1038/nprot.2013.143 PMC3969860

[64] Abu-Remaileh, M., Persky, N. S., Lee, Y., Root, D. E. & Kaelin, W. G. Jr. Total loss of VHL gene function impairs neuroendocrine cancer cell fitness due to excessive HIF2alpha activity. *Proc. Natl Acad. Sci. USA* **121**, e2410356121 (2024). 39320914 10.1073/pnas.2410356121 PMC11459182

[65] Dobin, A. et al. STAR: ultrafast universal RNA-seq aligner. *Bioinformatics* **29**, 15–21 (2013). 23104886 10.1093/bioinformatics/bts635 PMC3530905

[66] Patro, R., Duggal, G., Love, M. I., Irizarry, R. A. & Kingsford, C. Salmon provides fast and bias-aware quantification of transcript expression. *Nat. Methods* **14**, 417–419 (2017). 28263959 10.1038/nmeth.4197 PMC5600148

[67] Love, M. I., Huber, W. & Anders, S. Moderated estimation of fold change and dispersion for RNA-seq data with DESeq2. *Genome Biol.* **15**, 550 (2014). 25516281 10.1186/s13059-014-0550-8 PMC4302049

[68] Cornwell, M. et al. VIPER: visualization pipeline for RNA-seq, a Snakemake workflow for efficient and complete RNA-seq analysis. *BMC Bioinform.* **19**, 135 (2018). 10.1186/s12859-018-2139-9 PMC5897949 29649993

[69] Abu-Remaileh, M. et al. Targeting of HIF2-driven cachexia in kidney cancer. *Zenodo*10.5281/zenodo.14902967 (2025).

