# Publisher Correction: argeting of HIF2-driven cachexia in kidney cancer

**DOI:** 10.1038/s41591-026-04236-6

**Published:** 2026-01-28

**Authors:** Muhannad Abu-Remaileh, Laura A. Stransky, Nikita Bhalerao, Nitin H. Shirole, Qinqin Jiang, Eddy Saad, Marc Machaalani, Sean M. Vigeant, Hilina Woldemichael, Charles Xu, Jing Lu, Hairong Wei, Zhihong Liu, William Sun, Kei Enomoto, Toni K. Choueiri, Jason R. Pitarresi, Steven A. Carr, Namrata D. Udeshi, William G. Kaelin

**Affiliations:** 1 Department of Medical Oncology, Dana-Farber Cancer Institute, Harvard Medical School, Boston, MA USA; 2 Broad Institute of MIT and Harvard, Cambridge, MA USA; 3 Division of Hematology–Oncology, Department of Medicine, University of Massachusetts Chan Medical School, Worcester, MA USA; 4 Department of Medicine, Beth Israel Deaconess Medical Center, Harvard Medical School, Boston, MA USA; 5 NiKang Therapeutics, Wilmington, DE USA; 6 Howard Hughes Medical Institute, Chevy Chase, MD USA

**Keywords:** Renal cancer, Targeted therapies, Cancer metabolism

Correction to: *Nature Medicine* 10.1038/s41591-025-04054-2, published online 28 November 2025.

In the version of the article initially published, several figure legends contained incorrect units for the concentrations of PT2399 and doxycycline. Specifically, the legends for Fig. 2, Fig. 3, Extended Data Fig. 2, Extended Data Fig. 3, and Extended Data Fig. 6 incorrectly listed the concentration of PT2399 as 2 mM, whereas the correct concentration is 2 µM, as stated in the Methods. Additionally, the legend for Extended Data Fig. 5 incorrectly listed the doxycycline concentration as 1 mg/mL; the correct concentration is 1 µg/mL, also consistent with the Methods section. The legends are now amended in the HTML and PDF versions of the article.

