# Supplementary material for: Targeting of HIF2-driven cachexia in kidney cancer
Source: Nat Med. 2025 Nov 28;32(1):245–57. doi: 10.1038/s41591-025-04054-2 (PMC12823431; doi:10.1038/s41591-025-04054-2)
Supplement: Supplementary file 1 — Reporting Summary [file 41591_2025_4054_MOESM1_ESM.pdf]

nature portfolio

Corresponding author(s): William G. Kaelin, Jr.

Last updated by author(s): Mar 6, 2025

Reporting Summary

Nature Portfolio wishes to improve the reproducibility of the work that we publish. This form provides structure for consistency and transparency in reporting. For further information on Nature Portfolio policies, see our Editorial Policies and the Editorial Policy Checklist.

Statistics

For all statistical analyses, confirm that the following items are present in the figure legend, table legend, main text, or Methods section.

n/a	Confirmed	
☐	☒	The exact sample size (*n*) for each experimental group/condition, given as a discrete number and unit of measurement
☐	☒	A statement on whether measurements were taken from distinct samples or whether the same sample was measured repeatedly
☐	☒	The statistical test(s) used AND whether they are one- or two-sided<br>*Only common tests should be described solely by name; describe more complex techniques in the Methods section.*
☐	☒	A description of all covariates tested
☐	☒	A description of any assumptions or corrections, such as tests of normality and adjustment for multiple comparisons
☐	☒	A full description of the statistical parameters including central tendency (e.g. means) or other basic estimates (e.g. regression coefficient) AND variation (e.g. standard deviation) or associated estimates of uncertainty (e.g. confidence intervals)
☐	☒	For null hypothesis testing, the test statistic (e.g. *F*, *t*, *r*) with confidence intervals, effect sizes, degrees of freedom and *P* value noted<br>*Give P values as exact values whenever suitable.*
☒	☐	For Bayesian analysis, information on the choice of priors and Markov chain Monte Carlo settings
☒	☐	For hierarchical and complex designs, identification of the appropriate level for tests and full reporting of outcomes
☐	☒	Estimates of effect sizes (e.g. Cohen's *d*, Pearson's *r*), indicating how they were calculated

Our web collection on statistics for biologists contains articles on many of the points above.

Software and code

Policy information about availability of computer code

Data collection	Data collection was performed using standard laboratory equipment and commercially available software. Imaging analysis was conducted using ImageJ. RNA-seq data were collected using sequencing platforms following standard protocols. Mass spectrometry-based proteomics data were acquired and processed using Spectrum Mill.
Data analysis	GraphPad Prism v9 was used for statistical tests and data visualization.<br>R was used for statistical modeling and data processing. specifically, in Figure 3 and Extended Data Figure 8, 'tidyverse', 'dplyr' and 'ggrepel' to make the volcano plots, and in Figure 6 and Extended Data Figure 8, R 4.3.0 using Rstudio 2023.9.1.494 with the following packages: tidyverse 2.0.0, ggpubr 0.6.0, ggsci 3.0.0, and table1 1.4.3 have used for Belzutifan data.<br>SAS 9.4 software was used for NKT2152's data derivation and analysis.<br>Genomic and proteomic data processing:<br>STAR was used for RNA-seq alignment. DESeq2 was used for differential gene expression analysis. MACS2 was used for ChIP-seq peak calling. Spectrum Mill was used for proteomics data processing. The code used to analyze ChIP-seq, RNA-seq (24h and 48h PT2399 treatment), and PRO-seq datasets has been deposited in the Kaelin Lab GitHub repository and is publicly available at https://github.com/kaelinlabdfci/HIF2a_CCND1_ccRCC. The code used to analyze TCGA datasets has been deposited in the Kaelin Lab GitHub repository and is publicly available at https://github.com/kaelinlabdfci/HIF2-RCC-Cachexia.

For manuscripts utilizing custom algorithms or software that are central to the research but not yet described in published literature, software must be made available to editors and reviewers. We strongly encourage code deposition in a community repository (e.g. GitHub). See the Nature Portfolio guidelines for submitting code & software for further information.

Data

Policy information about availability of data

All manuscripts must include a data availability statement. This statement should provide the following information, where applicable:

- Accession codes, unique identifiers, or web links for publicly available datasets
- A description of any restrictions on data availability
- For clinical datasets or third party data, please ensure that the statement adheres to our policy

There are no restrictions on data availability for this study. The original mass spectra and protein sequence databases used for searches have been deposited in the public proteomics repository MassIVE under accession MSV000097181 (http://massive.ucsd.edu). The processed datasets, data tables from Fig. 2 and Fig. 3, Extended Data Fig. 3, the uncropped western blot scans, and the sex-stratified analyses of Belzutifan and NKT2152 clinical data in RCC patients for Fig. 6 and Extended Data Fig. 8 have been deposited in Zenodo and are publicly available at https://doi.org/10.5281/zenodo.17253773.
All raw and processed sequencing datasets have been deposited in GEO. RNA-seq (24h and 48h PT2399 treatment), ChIP-seq, and PRO-seq data are available under accession GSE277046 (https://www.ncbi.nlm.nih.gov/geo/query/acc.cgi?acc=GSE277046). RNA-seq (72h PT2399 treatment and sgEPAS1) data are available under accession GSE289579 (https://www.ncbi.nlm.nih.gov/geo/query/acc.cgi?acc=GSE289579). Polysome-seq data are available under accession GSE289581 (https://www.ncbi.nlm.nih.gov/geo/query/acc.cgi?acc=GSE289581).

Research involving human participants, their data, or biological material

Policy information about studies with human participants or human data. See also policy information about sex, gender (identity/presentation), and sexual orientation and race, ethnicity and racism.

Reporting on sex and gender	Sex was recorded from medical records based on sex assigned at birth and is reported in Extended Data Table 1 and Extended Data Table 2. Sex-disaggregated analyses for plasma PTHrP, corrected calcium, and body weight outcomes were performed and are presented in the rebuttal letter and described in the manuscript. No gender-related variables were relevant to the study design or interpretation.
Reporting on race, ethnicity, or other socially relevant groupings	Self-reported race and ethnicity were not collected.
Population characteristics	For the Belzutifan, ICI, and VEGFA TKI, the following clinical variables were collected and are reported in Table 1: age, sex, Eastern Cooperative Oncology Group (ECOG) performance status, clinical stage, number of prior lines of therapies, current treatment type, best response (RECIST 1.1), somatic VHL mutational status, as well as albumin-corrected calcium levels and body mass index at baseline, 1 month and 3 months after initiation of systemic therapy. For NKT2152 population characteristics are reported in Extended Data table 2: age, sex, Eastern Cooperative Oncology Group (ECOG) performance status, clinical stage, number of prior lines of therapies, current treatment type, best response (RECIST 1.1), as well as albumin-corrected calcium levels and body weight at start of therapy.
Recruitment	For the Belzutifan, ICI, and VEGFA TKI, this is a retrospective cohort collected from our institution (Dana-Farber Cancer Institute).For NKT2152, to be eligible for the trial (NCT05119335), patients had to be aged 18 years or older and with locally advanced or metastatic ccRCC and to have exhausted available standard therapy as determined by the investigator. 60 subjects were enrolled in the dose escalation part, among which 45 subjects had plasma samples for the PTHrP assay and data analysis.
Ethics oversight	For the Belzutifan, ICI, and VEGFA TKI, this study was approved by DFCIinstitutional review board (IRB) (#01-130). For NKT2152, the trial (NCT05119335) protocol and amendments were reviewed/approved by the appropriate ethic committees at respective participating sites. All patients provided written inform consent.

Note that full information on the approval of the study protocol must also be provided in the manuscript.

Field-specific reporting

Please select the one below that is the best fit for your research. If you are not sure, read the appropriate sections before making your selection.

☒ Life sciences ☐ Behavioural & social sciences ☐ Ecological, evolutionary & environmental sciences

For a reference copy of the document with all sections, see nature.com/documents/nr-reporting-summary-flat.pdf

Life sciences study design

All studies must disclose on these points even when the disclosure is negative.

Sample size	Sample sizes were determined based on previous studies and standard experimental designs. For mouse experiments, groups of 5–14 mice per condition were used, ensuring statistical power while minimizing animal use. For clinical data analysis, the sample sizes were based on available patient cohorts, as detailed in Tables 1 and 2.<br>No formal power calculation was performed, but the chosen sample sizes are sufficient to detect biologically relevant differences.

Data exclusions	No data were excluded from analysis unless pre-specified exclusion criteria were met. In clinical data analysis, patients with missing critical data points were excluded from specific statistical tests.
Replication	Experiments were independently replicated at least twice, with consistent results. RNA-seq, PRO-seq, and polysome-seq experiments were performed with biological triplicates to ensure reproducibility. Key findings were validated across multiple experimental conditions.
Randomization	Mice were randomly assigned to treatment groups upon reaching predefined tumor size thresholds or lossing > 10% of their body weight. For patient data analysis, randomization was not applicable since it was a retrospective observational study.
Blinding	Researchers were blinded to treatment groups during histological analysis and quantification of staining. Blinding was not possible during animal treatments due to practical constraints but was applied where feasible in data analysis. For NKT2153, no blinding in the trial (NCT05119335).

Reporting for specific materials, systems and methods

We require information from authors about some types of materials, experimental systems and methods used in many studies. Here, indicate whether each material, system or method listed is relevant to your study. If you are not sure if a list item applies to your research, read the appropriate section before selecting a response.

Materials & experimental systems

n/a	Involved in the study
☐	☒ Antibodies
☐	☒ Eukaryotic cell lines
☒	☐ Palaeontology and archaeology
☐	☒ Animals and other organisms
☐	☒ Clinical data
☒	☐ Dual use research of concern
☒	☐ Plants

Methods

n/a	Involved in the study
☐	☒ ChIP-seq
☒	☐ Flow cytometry
☒	☐ MRI-based neuroimaging

Antibodies

Antibodies used	Rabbit anti–Cyclin D1 (Cell Signaling Technologies; 2978), Rabbit anti-HIF2α (D6T8V) (Cell Signaling Technology; 29973S), Rabbit anti-HIF1α (D2U3T) (Cell Signaling Technology; 14179S), Rabbit anti-NDRG1(Cell Signaling Technology; 5196S), Mouse anti-Vinculin (Sigma; V9131), Rabbit anti-V5-Tag (Cell Signaling Technology; 13202S), Mouse anti-b-Actin (Cell Signaling Technology; 3700S), anti-streptavidin-HRP (Cell Signaling Technology; 3999S), Rabbit anti-IGFBP3 (Cell Signaling Technology; 25864S), Rabbit anti-MMP2 (D2O4T) (Cell Signaling Technology; 87809S), and Rabbit anti-Ucp1 (Sigma CAT #U6382). Unless otherwise noted, antibodies were used at 1:1000 in 5% BSA; anti-b-Actin was used at 1:2000.
Validation	All antibodies were validated by the manufacturer for the species and applications used in this study. Validation details, including specificity and application suitability, are available on the manufacturer's website and cited in previously published literature. Internal validation was performed by western blotting in relevant cell lines and tissues, confirming expected molecular weight and specific band detection.

Eukaryotic cell lines

Policy information about [cell lines and Sex and Gender in Research]

Cell line source(s)	OSRC-2 (from male) and RXF393 (from male) were obtained from the Riken Cell Bank and the National Cancer Institute (NCI), USA, respectively. SCC-9 (from male) and SCC-4 (from male) were obtained from ATCC. 786-O (from male) and 293T (from female) were obtained from the Kaelin's laboratory stocks at Dana-Farber Cancer Institute. The sex of these cell lines is not relevant to this study, as they were used for functional assays rather than sex-based comparisons.
Authentication	OSRC-2, RXF393, 786-O, 293T, SCC-4, and SCC-9 cell lines were authenticated by ATCC using STR (short tandem repeat) profiling in 2024-2025.
Mycoplasma contamination	All cell lines were routinely tested for mycoplasma contamination using a a MycoAlert Mycoplasma Detection kit (Lonza; LT07-318). All tested negative before experimental use.
Commonly misidentified lines (See ICLAC register)	No commonly misidentified cell lines from the ICLAC register were used in this study.

Animals and other research organisms

Policy information about studies involving animals; ARRIVE guidelines recommended for reporting animal research, and Sex and Gender in Research

Laboratory animals	Mouse models were used in this study. The strains included Nude (NCRNU-F sp/sp CrTac:NCr-Foxn1nu, Taconic) and NOD scid gamma (NOD.Cg-Prkdcscid Il2rgtm1Wjl/SzJ, NSG, Jackson Labs). Mice were 8–10 weeks old at the start of the experiments. All experiments followed institutional guidelines for animal care and use.
Wild animals	This study did not involve wild animals.
Reporting on sex	All mice used in this study were female. Female mice were chosen based on prior experience with the OSRC-2 xenograft model, which showed more consistent tumor engraftment and treatment response in females. Additionally, female mice are easier to handle and less aggressive than males, reducing variability due to handling stress. As only females were used, sex-disaggregated comparisons were not applicable and were not conducted.
Field-collected samples	This study did not involve field-collected samples.
Ethics oversight	All animal experiments were approved by the Institutional Animal Care and Use Committee (IACUC) at Dana-Farber Cancer Institute (protocol number 04-019) and the University of Massachusetts Chan Medical School (protocol number 202200072) .

Note that full information on the approval of the study protocol must also be provided in the manuscript.

Clinical data

Policy information about clinical studies
All manuscripts should comply with the ICMJE guidelines for publication of clinical research and a completed CONSORT checklist must be included with all submissions.

Clinical trial registration	For NKT2152, NCT05119335.
Study protocol	For NKT2152, NCT05119335 is posted at clinicaltrials.gov.
Data collection	For NKT2152, Clinical data (NCT05119335) was collected at participating sites that are all in the US.
Outcomes	N/A

Plants

Seed stocks	N/A
Novel plant genotypes	N/A
Authentication	N/A

ChIP-seq

Data deposition

☒ Confirm that both raw and final processed data have been deposited in a public database such as GEO.

☒ Confirm that you have deposited or provided access to graph files (e.g. BED files) for the called peaks.

Data access links<br>*May remain private before publication.*	https://www.ncbi.nlm.nih.gov/geo/query/acc.cgi?acc=GSE277046 by using the private token “afahaoqkflmhzsn”.
Files in database submission	OSRC2, Parental, Rep1<br>OSRC2, Parental, Rep2<br>OSRC2, HIF2a-KI, Rep1<br>OSRC2, HIF2a-KI, Rep2
Genome browser session<br>(e.g. UCSC)	N/A

Methodology

Replicates	Two Replicates
Sequencing depth	75 bp paired-end reads were sequenced on a NextSeq instrument
Antibodies	FLAG-M2 Sigma F1804
Peak calling parameters	Sequence tags were aligned with Burrows-Wheeler Aligner (BWA, RRID: SCR_010910) to build hg19 and uniquely mapped, non-redundant reads were retained. These reads were used to generate binding sites with Model-Based Analysis of ChIP-Seq 2 (MACS v2.1.1.20160309), with a q-value (FDR) threshold of 0.01. We evaluated multiple quality control criteria based on alignment information and peak quality: (i) sequence quality score, (ii) uniquely mappable reads (reads that could only map to one location in the genome), (iii) uniquely mappable locations (locations that could only be mapped by at least one read), (iv) peak overlap with Velcro regions, a comprehensive set of locations – also called consensus signal artifact regions – in the genome that have anomalous, unstructured high signal or read counts in next-generation sequencing experiments independent of cell line and of experiment type, (v) number of total peaks (each sample had >3,000 peaks, passing the minimum requirement of 1000), (vi) high-confidence peaks (the number of peaks that were enriched tenfold over the background), (vii) percentage overlap with known DHS sites derived from the ENCODE Project (samples meet the minimum 80% threshold), and (viii) peak conservation (a measure of sequence similarity across species based on the hypothesis that conserved sequences are more likely to be functional). All samples in this study passed quality control criteria. To identify HIF2-alpha peak location bed file was extended by 1 kb, 5 kb, 10 kb for each peak region using BEDOPS tool (RRID: SCR_012865).
Data quality	These reads were used to generate binding sites with Model-Based Analysis of ChIP-Seq 2 (MACS v2.1.1.20160309), with a q-value (FDR) threshold of 0.01. We evaluated multiple quality control criteria based on alignment information and peak quality: (i) sequence quality score, (ii) uniquely mappable reads (reads that could only map to one location in the genome), (iii) uniquely mappable locations (locations that could only be mapped by at least one read), (iv) peak overlap with Velcro regions, a comprehensive set of locations – also called consensus signal artifact regions – in the genome that have anomalous, unstructured high signal or read counts in next-generation sequencing experiments independent of cell line and of experiment type, (v) number of total peaks (each sample had >3,000 peaks, passing the minimum requirement of 1000), (vi) high-confidence peaks (the number of peaks that were enriched tenfold over the background), (vii) percentage overlap with known DHS sites derived from the ENCODE Project (samples meet the minimum 80% threshold), and (viii) peak conservation (a measure of sequence similarity across species based on the hypothesis that conserved sequences are more likely to be functional). All samples in this study passed quality control criteria.
Software	Integrative Genomics Viewer (https://software.broadinstitute.org/software/igv/ RRID: SCR_011793
