# Supplementary material for: Targeting of HIF2-driven cachexia in kidney cancer
Source: Nat Med. 2025 Nov 28;32(1):245–57. doi: 10.1038/s41591-025-04054-2 (PMC12823431; doi:10.1038/s41591-025-04054-2)

Fig. 1e

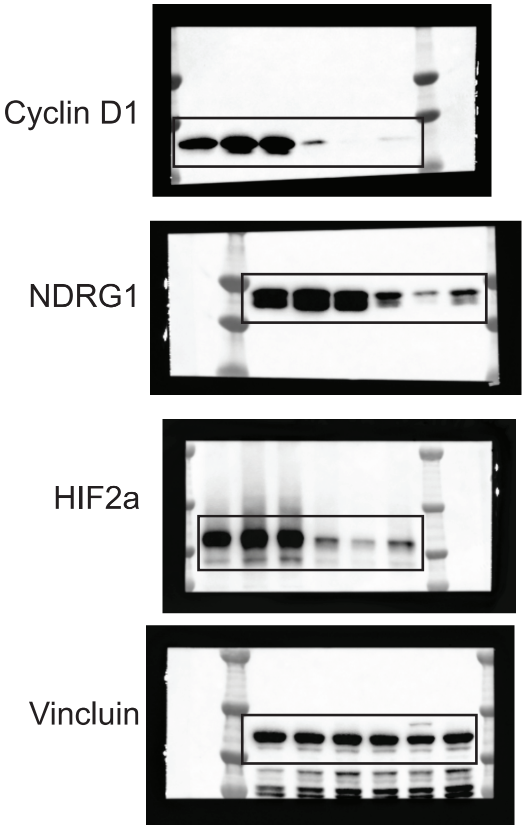

Fig. 2a,b

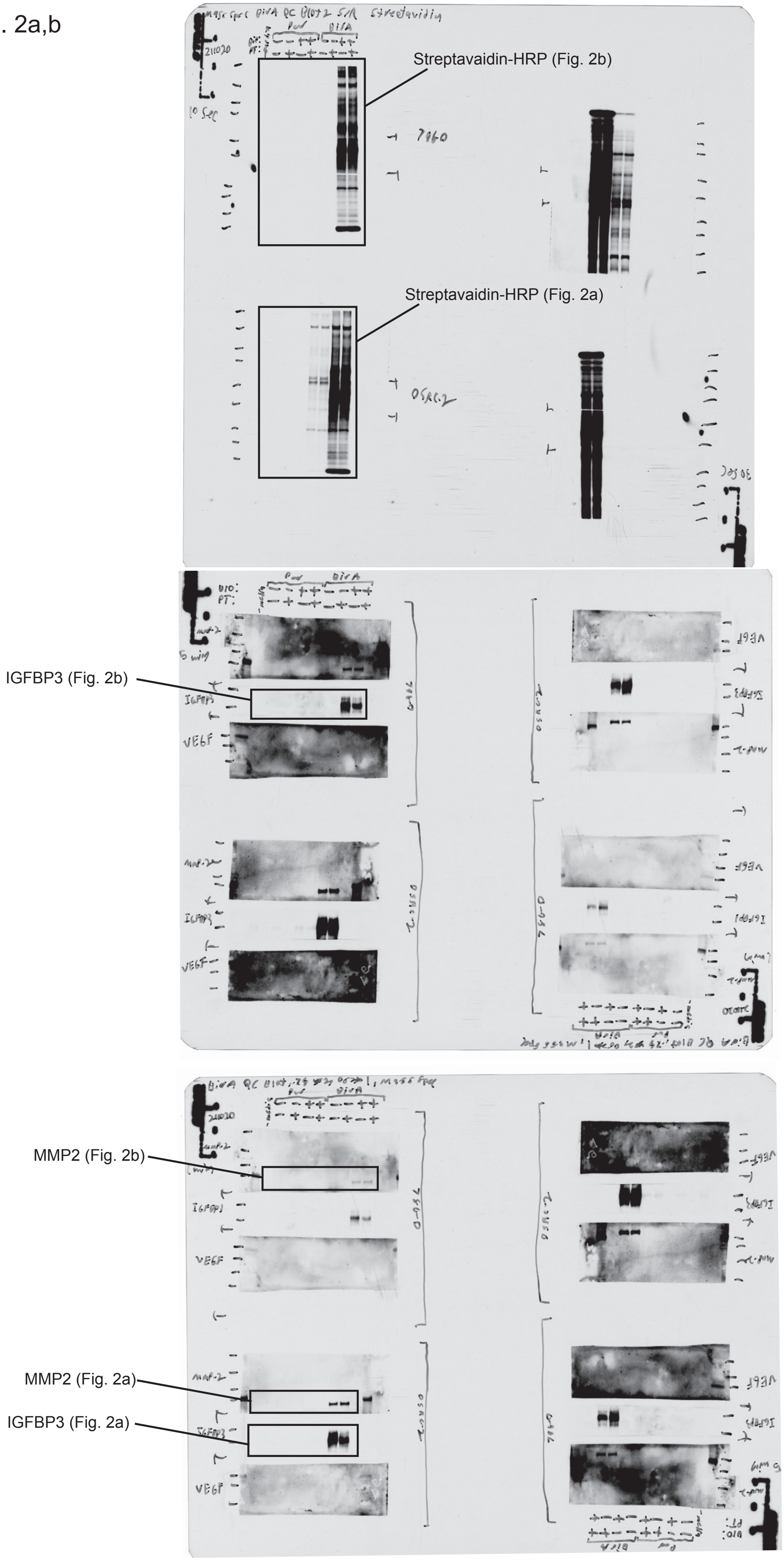

Streptavaidin-HRP (Fig. 2b)
Streptavaidin-HRP (Fig. 2a)
IGFBP3 (Fig. 2b)
MMP2 (Fig. 2b)
MMP2 (Fig. 2a)
IGFBP3 (Fig. 2a)

Extended Data Fig. 1g

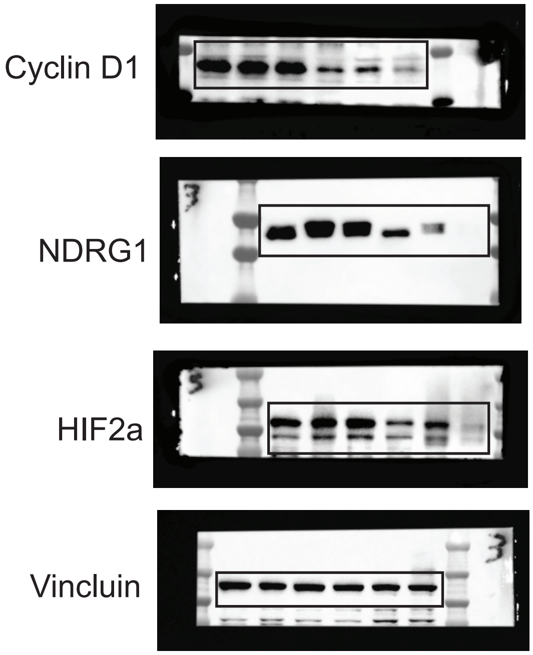

Extended Data Fig. 3e

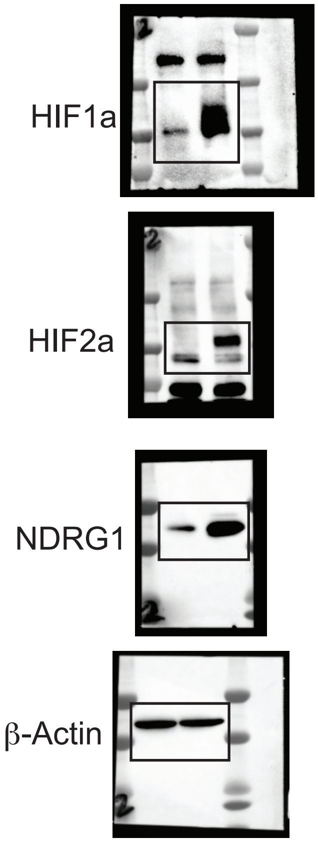

Extended Data Fig. 3i

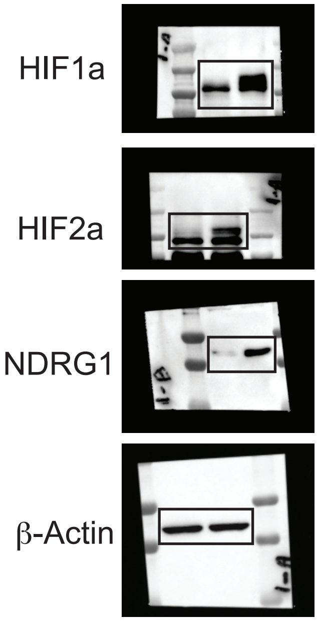

Extended Data Fig. 4d

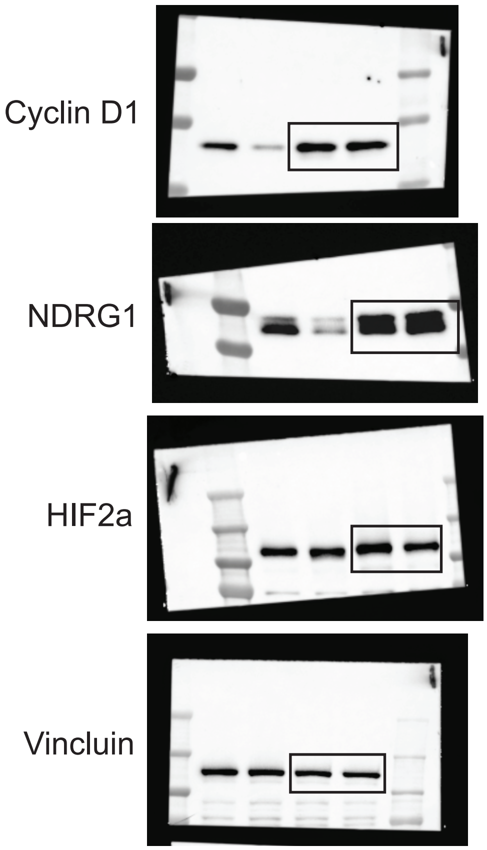

Extended Data Fig. 4e

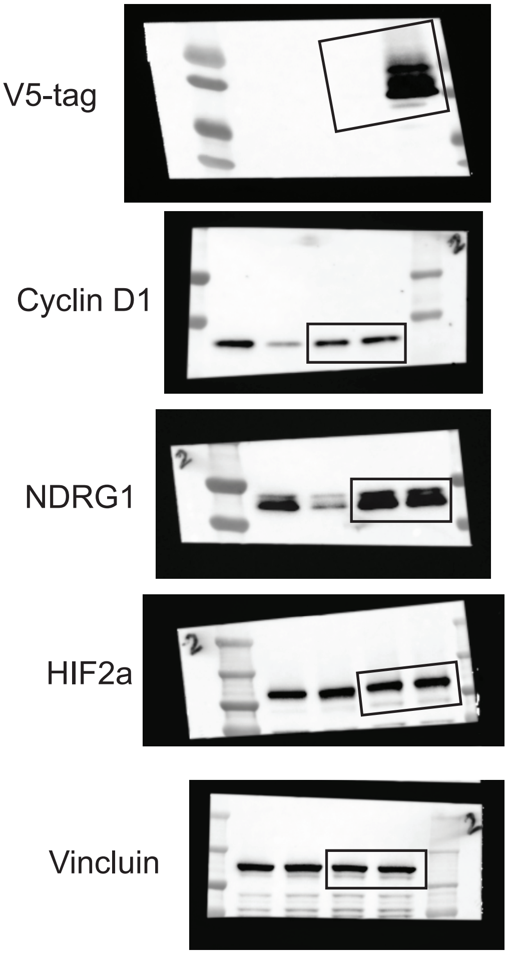

Extended Data Fig. 4g

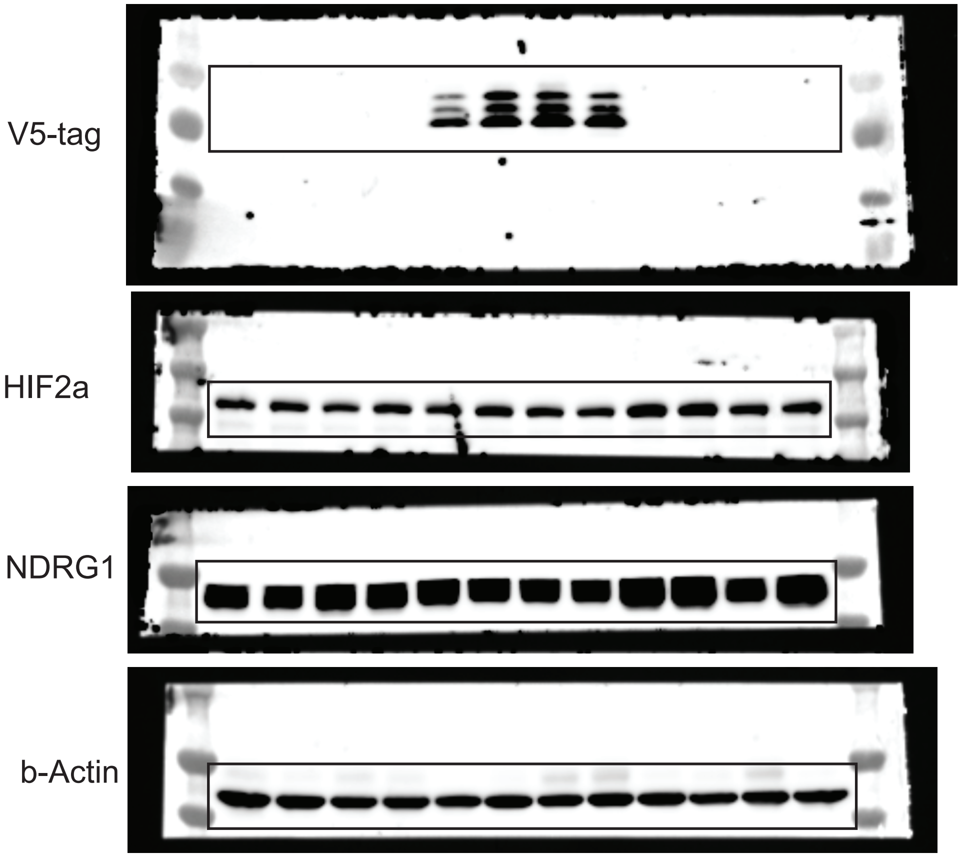

Extended Data Fig. 5a

V5-tag

Cyclin D1

NDRG1

HIF2a

Vincluin

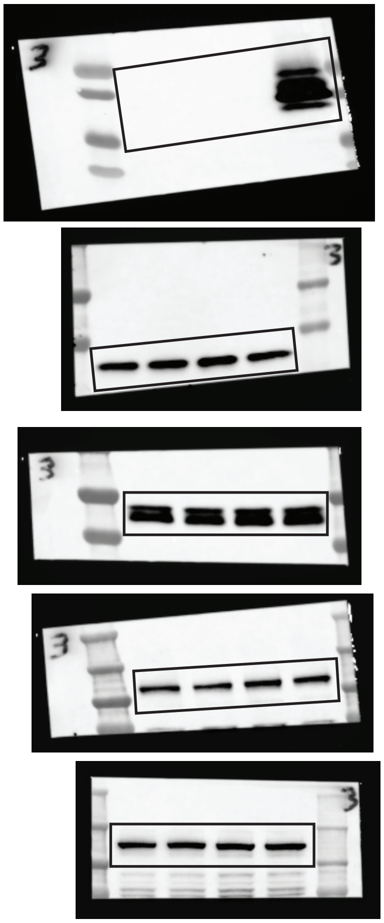

Extended Data Fig. 5d

V5-tag

NDRG1

b-Actin

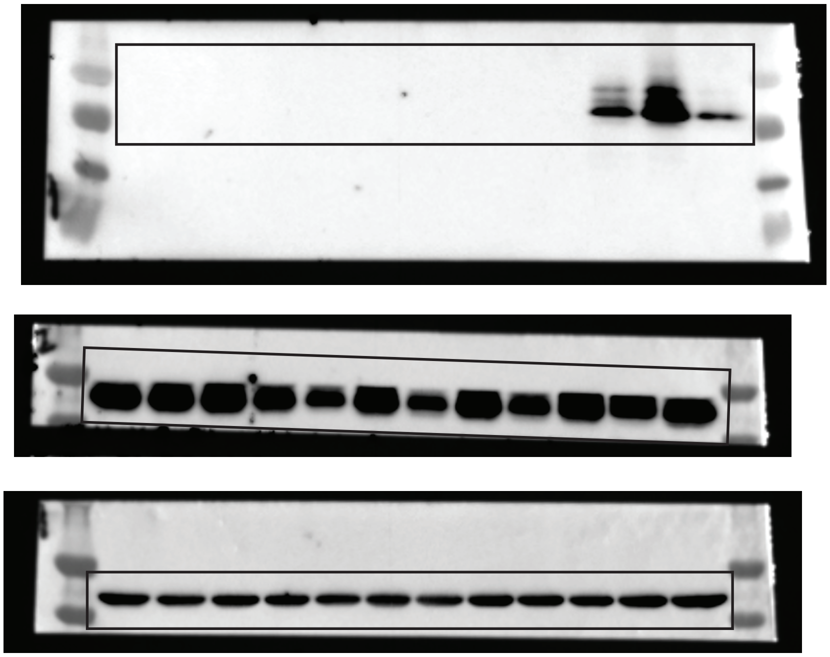

Extended Data Fig. 6h

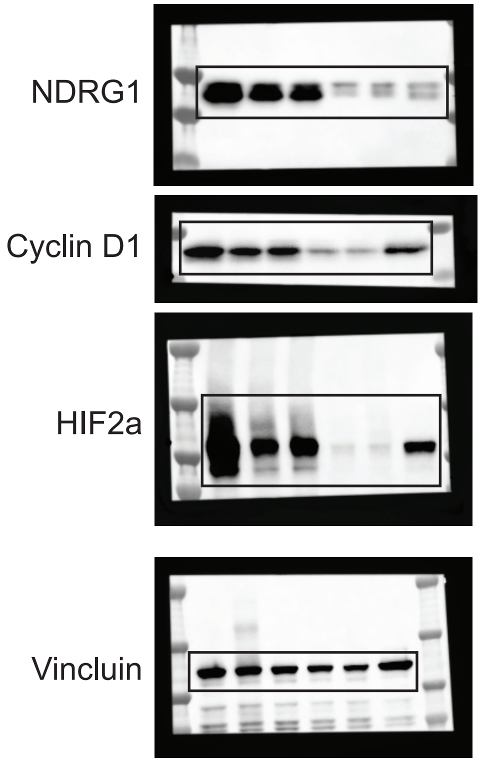

Supplement: Supplementary file 2 — Unprocessed western blots. [file 41591_2025_4054_MOESM2_ESM.pdf]
